# Ribozymes, DNAzymes, and Fluorescent Aptamers: Diversity, Mechanisms, and Prospects for Bioanalytical Applications

**DOI:** 10.3390/molecules31213861

**Published:** 2026-10-26

**Authors:** Elizaveta A. Shatunova, Mariya A. Vorobyeva, Svetlana V. Markova, Eugene S. Vysotski

**Affiliations:** 1 Institute of Biophysics of the Siberian Branch of the Russian Academy of Sciences, Federal Research Center “Krasnoyarsk Science Center” of the Siberian Branch of the Russian Academy of Sciences, Krasnoyarsk 660036, Russia; lizashatunova@yandex.ru (E.A.S.); eugene_vysotski@ibp.ru (E.S.V.); 2 Knorre Institute of Chemical Biology and Fundamental Medicine of the Siberian Branch of the Russian Academy of Sciences, Novosibirsk 630090, Russia

**Keywords:** ribozymes, DNAzymes, fluorescent aptamers, functional nucleic acids, nucleic acid catalysis, structure–function relationships, bioanalytical applications, biosensors, molecular imaging

## Abstract

Ribozymes, DNAzymes, and fluorescent light-up aptamers combine sequence-programmable recognition with catalytic activity or ligand-dependent signal generation. Our review examines their functional diversity, current progress in studies of structure and mechanisms of action, and potential for analytical applications. Natural and in vitro-selected ribozymes and DNAzymes are considered in relation to phosphodiester-bond hydrolysis, ligation, phosphorylation/dephosphorylation, covalent modification, photochemical reactions, and peroxidase-like oxidation. Particular attention is given to the relationship between catalytic core architecture, substrate and cofactor positioning, metal ion coordination, nucleobase participation, and reaction mechanisms. We consider fluorescent aptamer–fluorogen pairs as analogs of catalytic nucleic acids, since they employ RNA or DNA structures to activate fluorescence through fluorogen immobilization, conformational restriction, or chemical transformation. Bioanalytical applications of ribozymes and DNAzymes include the detection of nucleic acids, viral RNA, metal ions, nucleotides, enzymes, pathogens, and small molecules, while aptamer/fluorogen pairs are particularly well-suited for fluorescence imaging of RNA and metabolites in living cells. The review highlights the growing integration of catalytic nucleic acids with amplification strategies, nanomaterials, aptamer domains, and optical or electrochemical reporters. Although functional diversity increases almost every year, structural information and comprehensive structure–function datasets remain limited. Future progress will depend on structural characterization, improved computational modeling, machine-learning-assisted design, and the development of robust, biocompatible systems for broad bioanalytical use.

## 1. Introduction

In the early 1980s, it was demonstrated that nucleic acids (NA), primarily RNA, can serve not only as carriers and transmitters of genetic information but also as catalysts for chemical reactions. Later, it was shown that they are capable of specifically binding molecules of diverse chemical structures due to spatial complementarity. This led to the development of a distinct branch of molecular biology—the search for, synthesis, and study of catalytically active nucleic acids (ribozymes and DNAzymes), as well as aptamers, which act as highly specific molecular binders [1,2,3]. Thanks to the chemical nature of oligonucleotides, these types of functional nucleic acids can be combined, giving catalytically active RNA and DNA molecules with allosteric regulation [4].

The availability of diverse SELEX protocols and schemes has provided a large variety of artificial ribozymes and DNAzymes that can be chemically synthesized or expressed in living cells. In this review, we examine artificial catalytic nucleic acids together with fluorescent light-up aptamers. What conceptually links these two classes of functional oligonucleotides? Like ribozymes and DNAzymes, light-up aptamers produce a certain functional output—in their case, light emission. Once a fluorogenic ligand has bound, its ability to emit light is determined by precise conformation and positioning within the complex. This mechanistic parallel brings an important practical consequence: both classes can generate measurable analytical signals and therefore provide useful tools for bioanalysis and live cell imaging. Traditionally, these functional nucleic acids have been generated by means of in vitro selection. The rapid development of computational methods, however, attracts increasing attention to the possibility of using in silico approaches: not necessarily to replace experimental selection altogether, but at least to improve and refine catalytic nucleic acids and light-up aptamers. In general, is the field ready for such a shift in strategy? More specifically, can the available evidence sufficiently answer the following questions: How do nucleic acid structure, substrate or fluorogen positioning, nucleobase chemistry, and metal ion or cofactor interactions determine catalytic or signal-generating function, and can these relationships guide bioanalytical design?

Most recent reviews discuss catalytic nucleic acids and light-up aptamers separately or cover them within broader surveys of functional nucleic acids [3,5,6,7,8]. A notable exception is the 2020 review by Micura and Höbartner [2], which analyzed catalytic nucleic acids, fluorogenic aptamers, and riboswitches from structural and functional perspectives. However, it covers only a selective range of nucleic acid catalysts. Here, we review a broader spectrum of nucleic acid-catalyzed reactions to reveal general trends in the field and assess both their strengths and limitations. We also give some representative examples for bioanalytical applications of catalytic nucleic acids and light-up aptamers. They illustrate the practical value of both classes and emphasize the necessity of more rational, structure-guided strategies for their development.

## 2. Ribozymes and DNAzymes

The vast majority of catalytic RNAs within living organisms catalyze the cleavage of phosphodiester bonds by intramolecular transesterification. An alternative example is the peptidyl transferase activity of ribosomal RNA. As yet, no examples of naturally occurring catalytic DNA have been reported. Otherwise, in vitro selection technology allowed creating a whole range of artificial catalytic NAs with different activities. While the active site structure and catalytic mechanisms of natural ribozymes have been studied in great detail, structural studies of artificial catalytic nucleic acids are the exception rather than the rule. Table 1 summarizes the data on established or at least hypothesized catalytic mechanisms for artificial ribozymes and DNAzymes.

In general, we should mention that the entire body of articles on artificial catalytic nucleic acids is highly heterogeneous, so they are quite difficult to classify. Researchers often prioritize the discovery of new catalytic activities through SELEX, whereas the structural and mechanistic basis of the resulting catalysts receives less attention. This heterogeneity has, to some extent, shaped the organization and presentation of the material in this review. Here, we used the type of chemical reaction as a main principle of classification, but in some cases catalytic NAs were grouped on the basis of their functionality.

### 2.1. Natural Ribozymes

**Figure 1:**
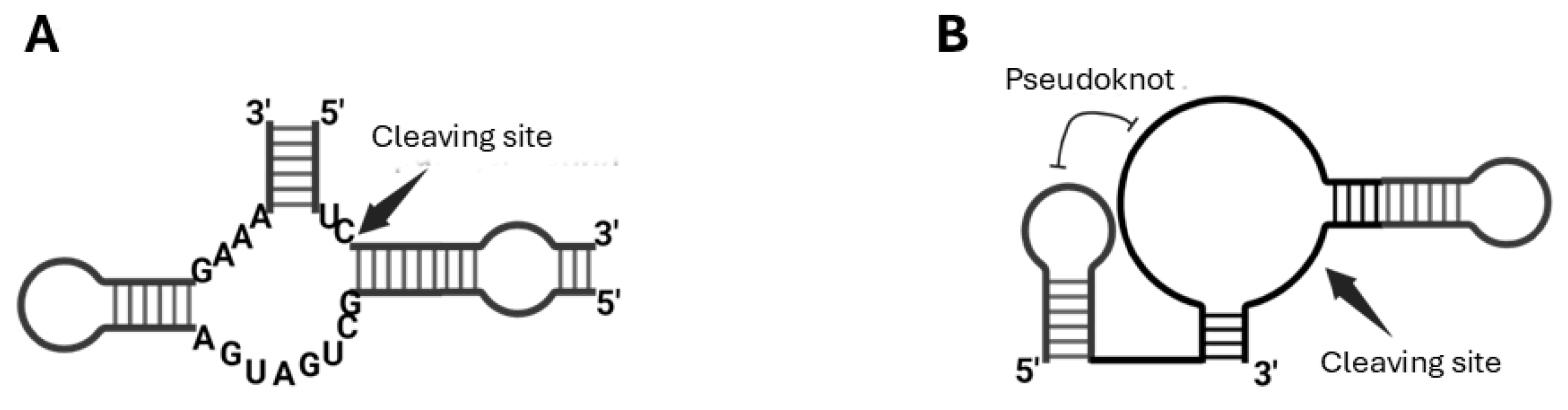
A schematic representation of the *trans*-hammerhead ribozyme (**A**) and the pistol ribozyme (**B**).

Since the discovery of ribozyme catalysis, the cleavage of phosphodiester bonds remains an important topic in the chemistry of catalytic nucleic acids [9]. Several classes of natural ribozymes perform site-specific intramolecular transesterification: the hepatitis delta virus ribozyme (HDVr), the Varkud satellite ribozyme (VSr), the hairpin ribozyme (Hpr), the hammerhead ribozyme (Hhr) (Figure 1A), and the glmS ribozyme [2,10,11,12,13,14,15]. Later, bioinformatic methods expanded this diversity with the twister (Twr), twister sister (Twsr), pistol (Plr) (Figure 1B), and hatchet (Hcr) ribozymes [9,13], which likewise catalyze site-specific cleavage of the 3′–5′-phosphodiester bond and internal phosphoester transfer via the Sn2 mechanism, giving a 2′,3′-cyclophosphate [2,16,17,18].

**Figure 2:**
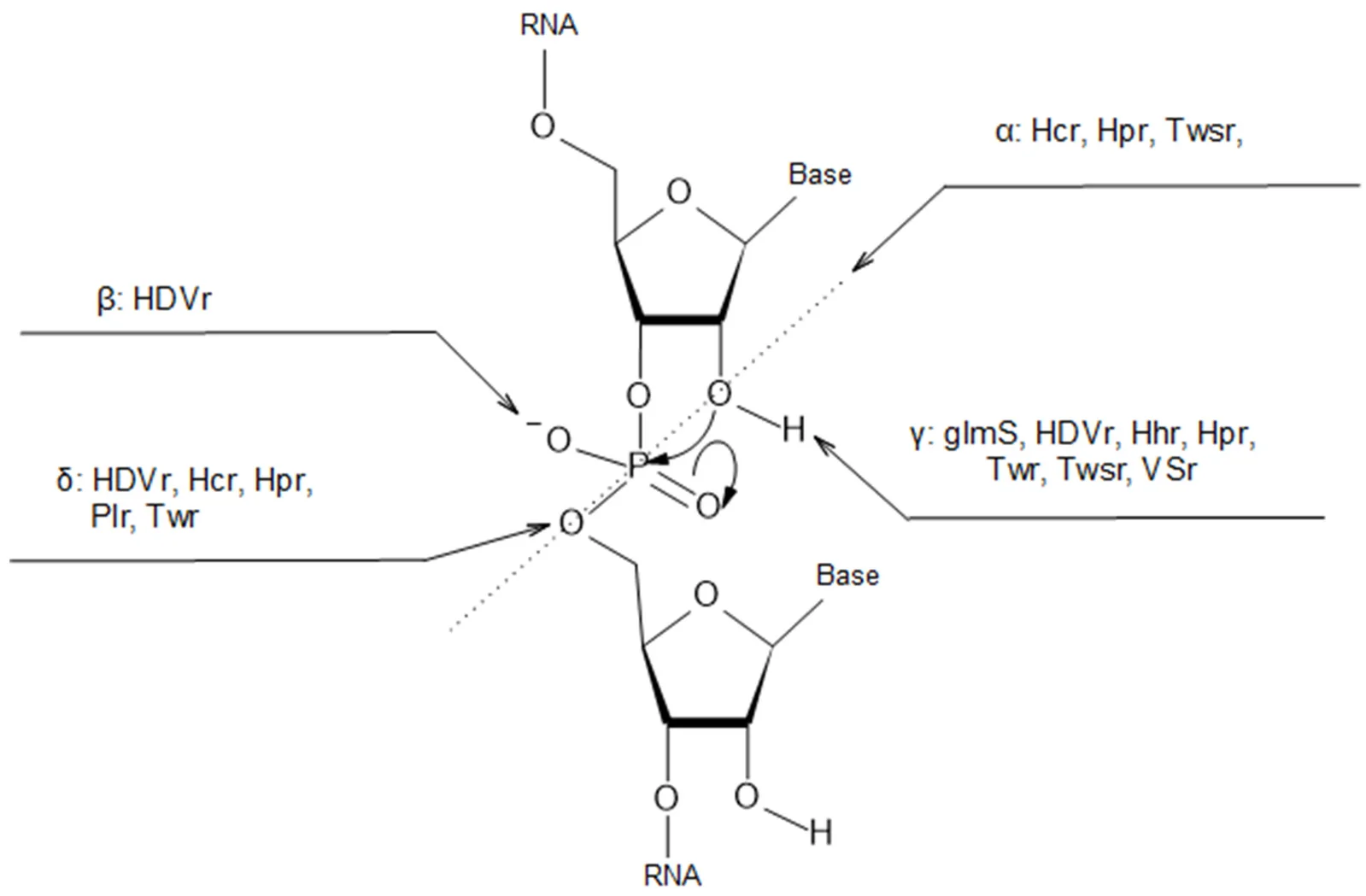
Strategies for catalyzing the cleavage of phosphodiester bonds by natural self-cleaving ribozymes.

The acid–base mechanism relies on a combination of the four strategies, each contributing to a different degree depending on the ribozyme type [2,13,17,19,20] (Figure 2):

- Linear positioning of 2′-oxygen, phosphorus, and 5′-oxygen for a nucleophilic attack (α-catalysis);
- Electrostatic compensation of the negative charge on nonbridging phosphate oxygen (β-catalysis);
- Deprotonation of the attacking 2′-OH-nucleophile (general base catalysis, γ-catalysis);
- Neutralization of the negative charge forming at the 5′-O by a donor proton (general acid catalysis, δ-catalysis).

For the twister ribozyme (Twr), the main contributions come from γ-catalysis (deprotonation of the 2′-OH nucleophile with involvement of guanine) and δ-catalysis (N3 of adenine in the active site acts as a proton donor for the leaving 5′-OH group) [21]. In the case of pistol ribozyme (Plr), the hydrated Mg^2+^, coordinated to N7 of guanine, stabilizes the leaving 5′-OH group (δ-catalysis) [13,22,23,24]. Hammerhead, hatchet, and twister sister ribozymes use the 2′-OH group deprotonation (γ-catalysis) facilitated by guanine in the active site or Mg^2+^; α-catalysis makes an additional contribution in the case of Hcr and Twsr [13,22,25,26,27,28,29]. Adenine and guanine in the active site of the hairpin (Hpr) ribozyme function as an acid and base (γ- and δ-catalysis) as the main mechanism, while in-line orientation in the active site also plays a significant role (α-catalysis) [17,29,30,31]. In the case of the hepatitis delta virus ribozyme (HDVr), the cytidine in the active site first deprotonates the 2′-OH group (γ-catalysis), then acts as a proton donor for the leaving OH group (δ-catalysis), while the Mg^2+^ cation stabilizes the transition state by compensating for the negative charge (β-catalysis) [29,32,33,34]. The Varkud satellite virus ribozyme (VSr) relies mainly on γ-catalysis [16,17,35,36,37]. The glmS ribozyme deserves special mention, as it utilizes a cofactor, glucosamine-6-phosphate (GlcN6P), which activates the 2′-OH nucleophile as a base (γ-catalysis); the other strategies make a minor contribution [29,36,38,39]. The possible combinations of catalytic strategies are summarized in Table 2.

Some self-cleaving cis ribozymes (Hhr, HDVr, Hpr, and glmS) can be split into two separate molecules (the so-called trans variant): a catalytic domain and a “substrate” with a cleavable bond [5,9]. This approach enables the specific cleavage of RNA within a selected nucleotide sequence and makes it possible to design synthetic ribozymes to inhibit gene expression at the mRNA level [5]. Furthermore, a combination with an aptamer domain supplies them with an additional option of activity regulation [5,9,40].

**Table 2:** Naturally occurring catalytic RNAs.

Class	Reaction	Catalytic Mechanism	Ref.
Twister	Site-specific cleavage of the 3′-5′-phosphodiester bond in RNA by internal transesterification with the formation of 2′,3′-cyclophosphate	γ, δ	[21]
Twister sister		γ	[28]
Pistol		δ	[23]
Hatchet		γ, α	[25]
Hammerhead		γ	[14]
Hairpin		γ, δ, α	[15]
HDVr		γ, δ, β	[33]
VSr		γ	[35]
glmS		γ	[38]
Ribonuclease P	Hydrolysis of the 3′-5′-phosphodiester bond in RNA with the formation of 5′-phosphate and 3′-OH	One Mg^2+^ activates the H_2_O molecule for nucleophilic attack; a second Mg^2+^ stabilizes the transition state and ensures the transfer of H^+^ to 3′-OH	[20,41,42]
Group I introns	Cleavage and ligation of the 3′-5′-phosphodiester bond in RNA	Two-step trans-esterification involving free guanine	[41,43,44]
Group II introns		Two-step trans-esterification involving adenine within the intron	
Ribosomal RNA	Peptidyl transferase reaction	Acid–base catalysis	[20,45]

The data on the structure of natural self-cleaving ribozymes and mechanisms of internal phosphoester transfer [11,16,17,18,22] established the basis for research of the structural features of other functional nucleic acids. For instance, the unique example of catalytic RNA is ribosomal RNA (23S in prokaryotes and 28S in eukaryotes). It facilitates the peptidyl transferase reaction in the cognate center of the ribosome during polypeptide chain synthesis by an acid–base mechanism involving (in eukaryotes) the 2′-OH group of A2451 and also C2452, U2506, U2585, A2602, and C2063 [45,46,47,48].

### 2.2. Artificial Ribozymes and DNAzymes

#### 2.2.1. In Vitro Selection of Catalytic Nucleic Acids

Artificial ribozymes and DNAzymes are created by in vitro selection, also known as SELEX (Systematic Evolution of Ligands by Exponential Enrichment). Basically, the technology includes targeted selection of molecules with desirable catalytic activity from combinatorial RNA or DNA libraries followed by the amplification of the enriched pool that enters the next round of selection.

**Figure 3:**
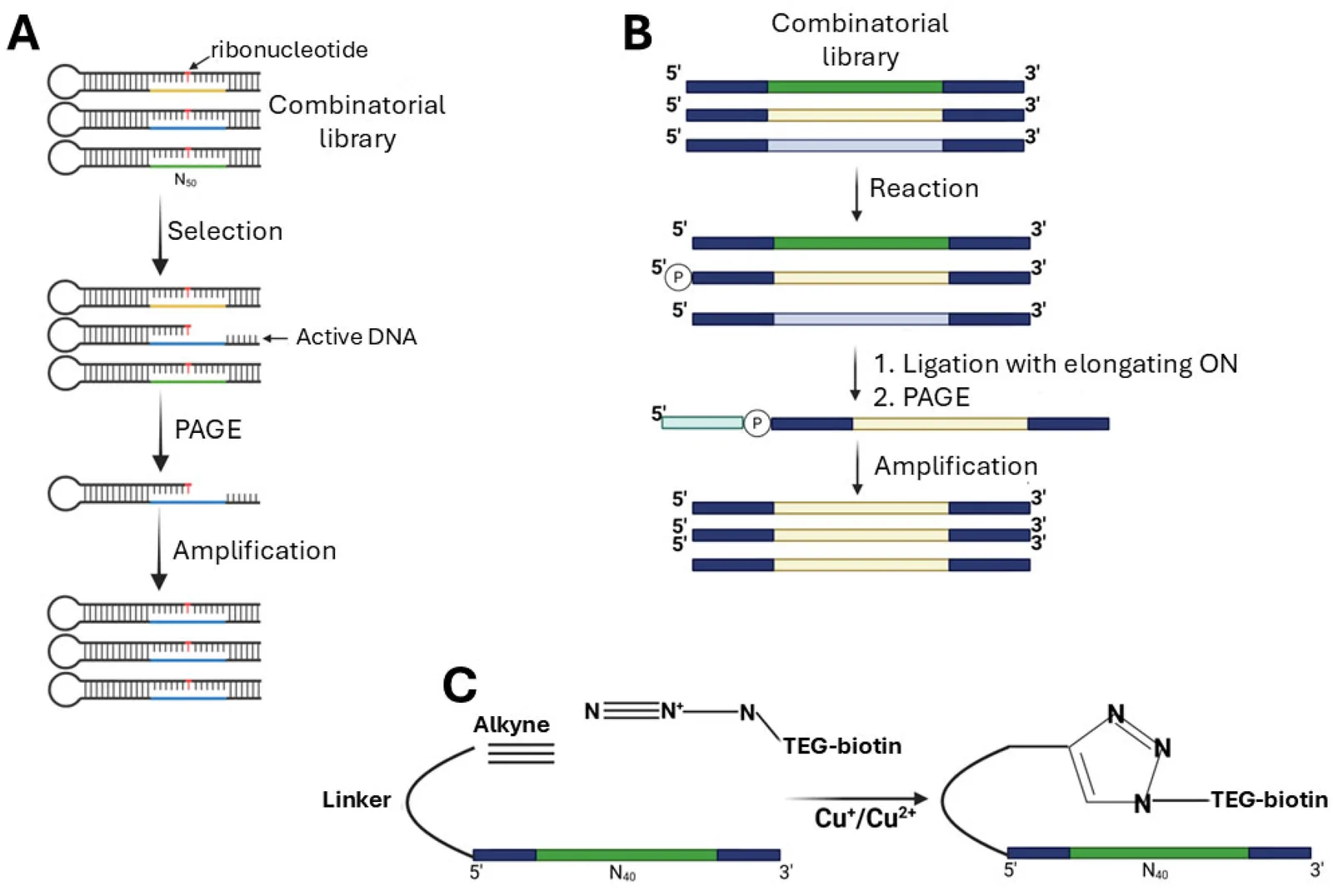
Strategies for isolating a pool of catalytically active NA during the in vitro selection of: RNA-cleaving DNAzymes (**A**); DNAzymes that catalyze phosphate transfer to their 5′-terminus (**B**), and DNAzymes that catalyze the CLICK reaction (**C**).

The cornerstone of the selection process is the proper positioning of the reaction substrate relative to the randomized part of the library. For this purpose, one can use additional oligonucleotides complementary to constant regions of the library or attach the substrate as a covalent terminal modification of a hairpin-forming nucleic acid. The separation of the pool of the active molecules from the rest of the library is no less important. Typically, the desired reaction modifies the selected nucleic acids, making them either shortened (Figure 3A) or extended (Figure 3B) for subsequent PAGE separation. Alternatively, they can be biotinylated and separated with a streptavidin-modified carrier (Figure 3C).

Some researchers further improve the efficacy of the selection by using a doped combinatorial library. The latter is derived from a particular RNA or DNA sequence obtained in the first selection step. Each position within the randomized part contains 70–90% of the nucleotide corresponding to a parent sequence and 10–30% of all other three nucleotides in equal proportions [2,49,50,51,52,53,54]. Such a ‘narrowed’ library allows for fine-tuning of certain positions during the selection process for better binding or catalysis.

For the purposes of this review, we used the type of chemical transformation catalyzed by nucleic acids as the primary classification criterion. Mechanistically distinctive systems (photochemical and peroxidase) and a large group of catalytic nucleic acids related to the framework of the RNA World hypothesis are highlighted separately.

#### 2.2.2. Phosphodiester Bond Cleavage

The first example of artificially selected catalytic DNAs, DNAzyme GR5, cleaves RNA in the presence of Pb^2+^ ions [55]. The general strategy for the selection of metal-dependent RNA-cleaving DNAzymes employs the insertion of a single ribonucleotide into a certain position of a combinatorial ssDNA library. After cleavage, the reacted molecules can be separated by PAGE (Figure 3A).

**Figure 4:**
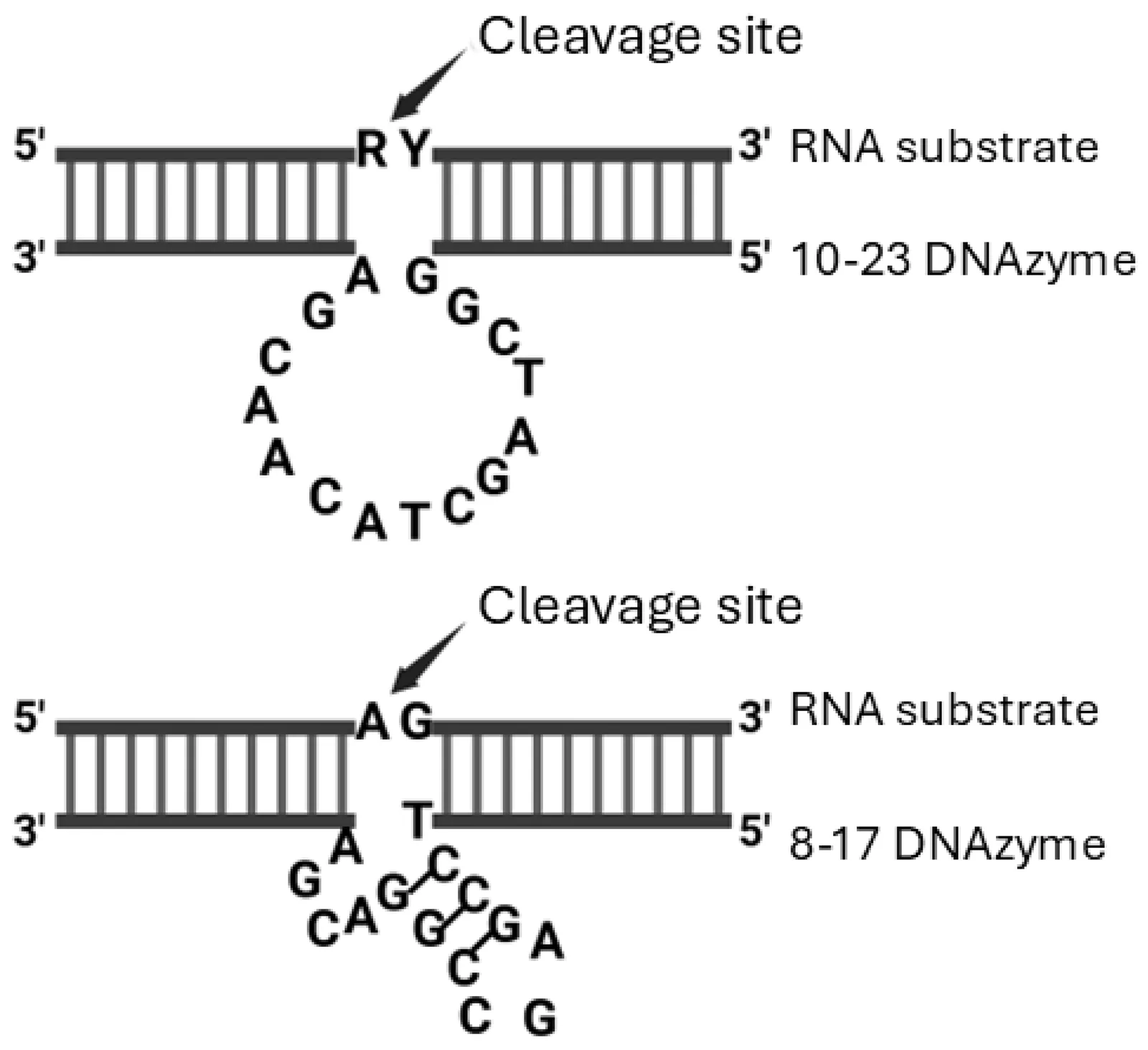
The catalytic cores of DNAzymes 10-23 and 8-17.

Other examples of RNA-cleaving DNAzymes include Mg^2+^-dependent DNAzymes 10-23 and 8-17 [56] (Figure 4), Na^+^-dependent DNAzymes [57], and DNAzymes that require other metal cations, L-His, or other low-molecular-weight compounds [51,58,59]. DNAzymes 8-17 and 10-23 use similar catalytic strategies, forming a pocket within the catalytic center to bind a divalent metal cation. The guanine in the active site acts as a base to activate the nucleophilic 2′-OH group, while the metal cation stabilizes the leaving group. In the case of DNAzyme 8-17, the architecture of the active site is based on a pseudoknot structure involving a non-canonical A-G Hoogsteen base pair [60,61,62,63].

Several examples of DNA-cleaving catalytic NAs were also generated: Cu^2+^-dependent DNAzyme [64], Zn^2+^-dependent DNAzyme [65], and sequence-specific Zn^2+^- and Mn^2+^-dependent DNAzyme [66].

#### 2.2.3. Phosphodiester Bond Formation

In nature, the mechanism of ribozyme-catalyzed RNA ligation is the reverse of that of hydrolysis [2,20]. Artificial RNA ligases catalyze as well both hydrolysis and the formation of a phosphodiester bond. Robertson and Joyce [67] described a self-replicating ribozyme, catalyzing the ligation of RNA fragments. It then became a basis for a series of shorter ligating RNAs [68,69,70].

DNAzyme ligases that catalyze 2′-5′ and 3′-5′ RNA ligation [71,72,73,74] act via an acid–base mechanism involving two Mg^2+^ ions for activation of the nucleophile and stabilization of the leaving group. An investigation into the crystal structure of 9DB1 DNAzyme revealed that the DNAzyme active site forms a double pseudoknot that also involves the RNA substrate, with the dT29–dT30 pair localized near the ligation site [75,76,77,78].

#### 2.2.4. Phosphoryl-Transfer Reactions

Ribozymes and DNAzymes that catalyze a phosphate group transfer are of special interest because of their clear potential for practical applications. The ability to perform phosphorylation/dephosphorylation makes them useful tools for regulating cellular processes and for developing bioanalytical systems.

The studies [79,80,81] describe the selection of ribozymes with polynucleotide kinase-like activity, transferring a γ-phosphate group from ATP either to their own 2′- or 5′-hydroxyl or to a separate RNA substrate. Burke’s group reported [82,83,84,85,86] the ribozyme K28 and its variants, which catalyze the transfer of thiophosphate to the ribose 2′-OH group. They proposed a three-stage reaction scheme; however, there is yet no data on the structure of the active site or the precise mechanism. Müller and co-workers examined the possibility of RNA-mediated catalysis for biomolecule synthesis by screening for ribozymes that catalyze the GTP synthesis from a nucleoside and a triphosphate or the triphosphorylation of the 5′-hydroxyl group of RNA [87,88,89].

Li and Breaker [90] developed DNAzyme-based polynucleotide kinases that phosphorylate a DNA substrate. Mn^2+^-dependent DNAzymes reported in [91,92,93,94] catalyze the transfer of phosphate from ATP or GTP to their own 5′-terminus. A DNAzyme that transfers a phosphate from the 5′-terminus of an RNA molecule to the DNA 3′-terminus is described in [95].

**Figure 5:**
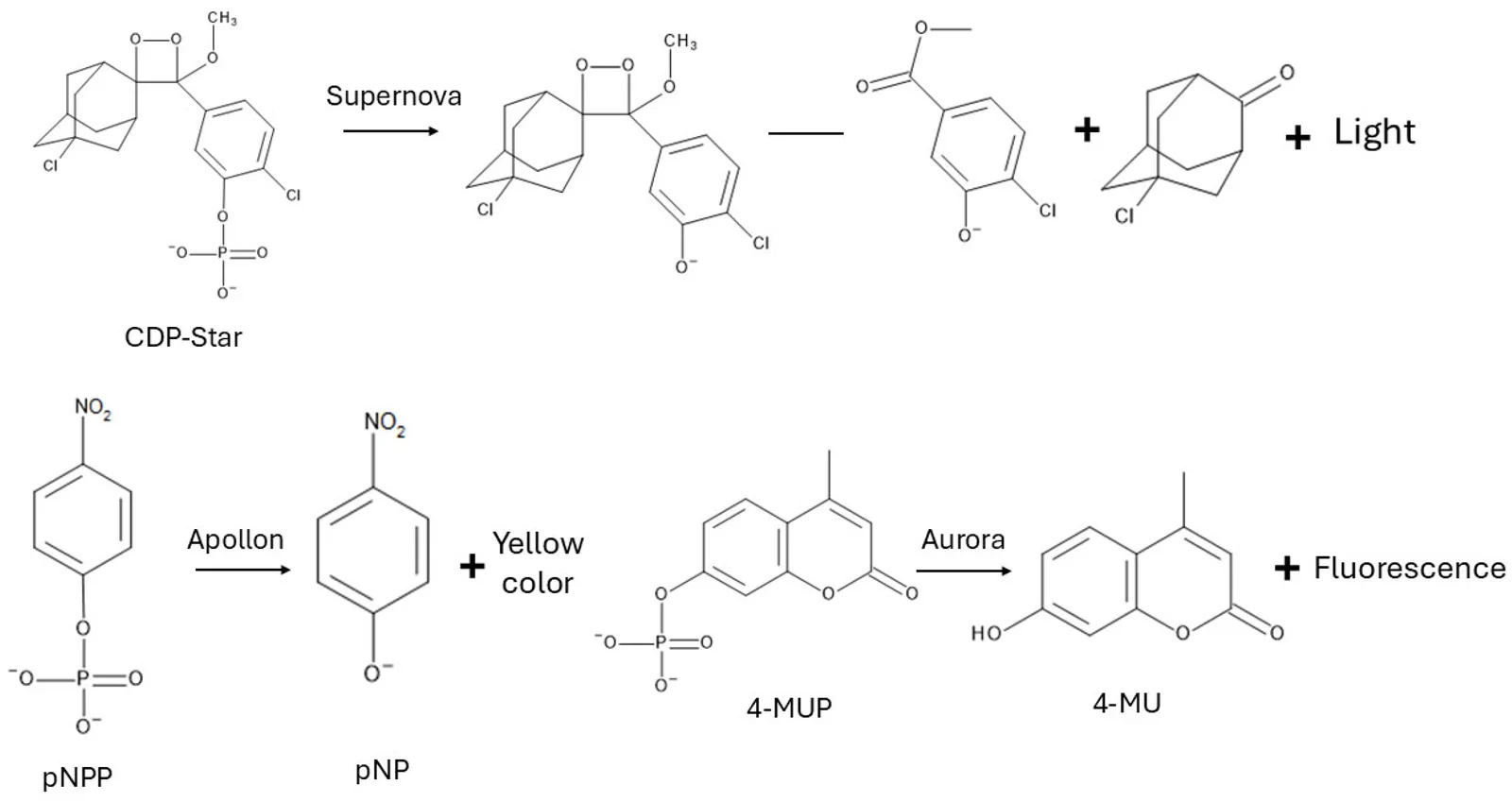
Reaction schemes for the dephosphorylation of commercial substrates by the Supernova DNAzyme family.

Recently, Curtis and co-workers produced Supernova, the Zn^2+^-dependent DNAzyme that autophosphorylates the 5′-OH group, transferring the phosphate from the CDP-Star, a commercially available chemiluminescent substrate for alkaline phosphatase [96,97]. The unstable dephosphorylation product degrades with the light emission (Figure 5). During the selection, the ssDNA library was incubated with the substrate, then the pool of 5′-phosphorylated DNAs was ligated to an elongating oligonucleotide and separated by PAGE (Figure 3B). The resulting DNAzyme showed itself to be a promising non-protein analog of alkaline phosphatase. Later, it became the basis of a doped library for the selection of other phosphatase-like DNAzymes. For example, the work [98] describes the production of the Apollon DNAzyme, which dephosphorylates p-nitrophenyl phosphate (pNPP), a commonly used colorimetric substrate (Figure 5). The DNAzyme Aurora obtained using a similar approach [99] dephosphorylates 4-methylumbelliferyl phosphate (4-MUP), giving a fluorescent signal (Figure 5).

As an example of another type of phosphatase-like DNAzyme, we should mention the DNAzyme produced by Silverman’s group [100], which dephosphorylates tyrosine and retains the activity even in the cell lysate.

#### 2.2.5. Other Covalent Modifications of Nucleic Acids

In vitro selection of a ribozyme that performs self-biotinylation via the formation of a C–N bond [101] has broadened the range of reactions accessible to catalytic nucleic acids. Subsequent studies provided a broad range of other alkylating ribozymes. For example, the paper [102] describes the selection of a ribozyme catalyzing the self-alkylation with fluorescein iodoacetamide (FIA): after incubation of the RNA library with FIA and precipitation, the reacted molecules were immunoprecipitated with anti-FIA antibodies on magnetic beads. The ability of the ribozyme to incorporate a fluorescent label into its structure might be used for labeling RNA within a cell.

**Figure 6:**
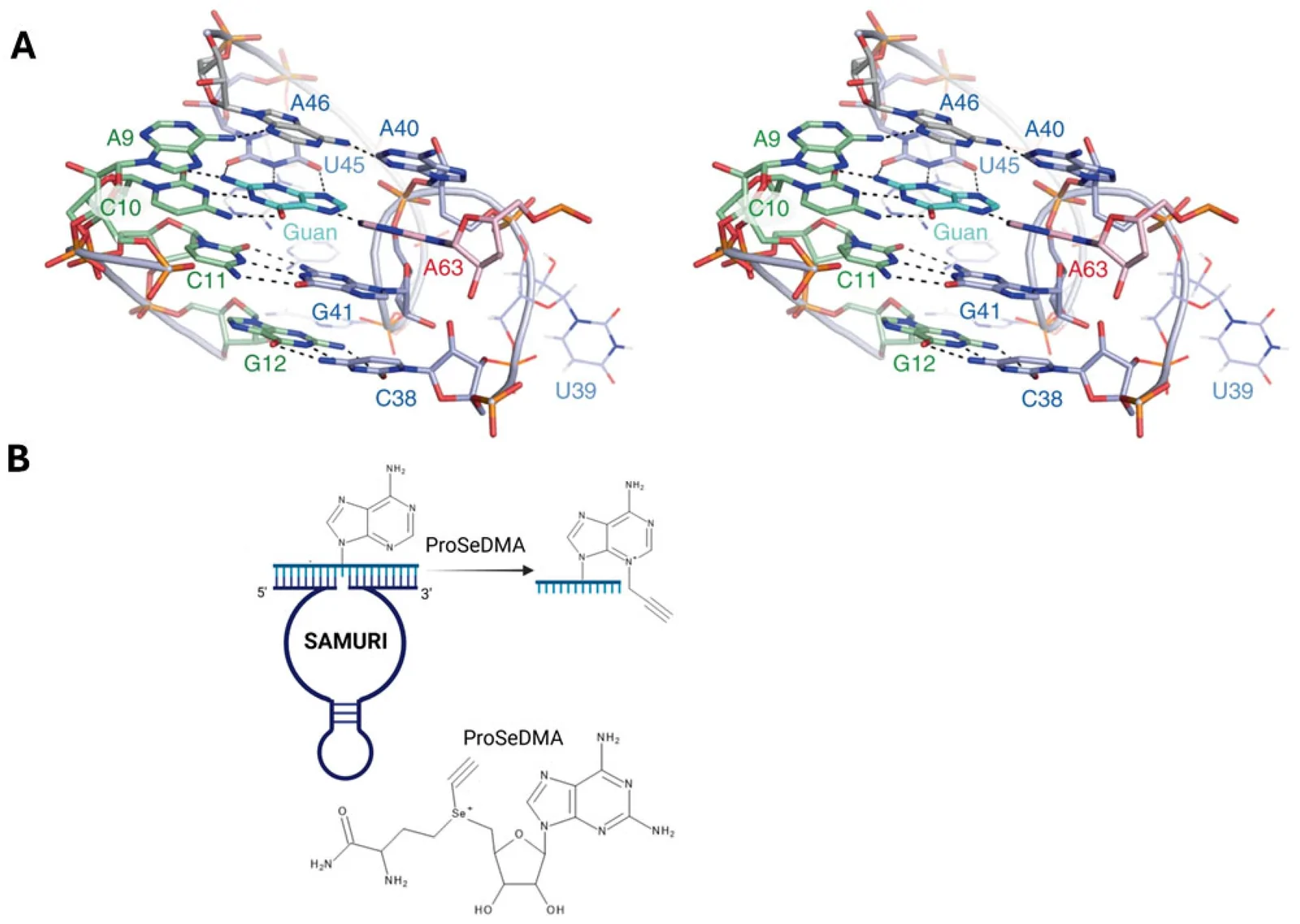
Ribozymes with methyltransferase and alkylating activity. (**A**) structure of the active site of the MTR1 ribozyme, from [104]; (**B**) reaction catalyzed by the SAMURI ribozyme.

The selection, structure, and mechanism of action of the methyltransferase MTR1 ribozyme are described in [103,104,105]. The ribozyme employs O6-methylguanine as a cofactor to catalyze site-specific methylation of adenosine at the N1 position. Within the three-way junction structure, C10 and U45 coordinate O6-methylguanine and align it for nucleophilic attack by N1 of A63 (Figure 6A). Another ribozyme with methyltransferase activity [106], a 33-nucleotide RNA, uses a S-adenosylmethionine cofactor (SAM) for site-specific methylation of the guanine N7, like natural methyltransferases. The alkylating ribozyme SAMURI [107,108] engages a synthetic SAM analog, ProSeDMA (propargylated Se-2,6-diaminopurine-selenomethioninamide), to propargylate N3 of adenosine of the RNA substrate (Figure 6B). SAMURI also forms a three-way junction where the C13-G33 pair and G30 organize the stacking-dependent structure of the active site. The C11-G36 pair provides the positioning of the substrate adenosine residue, G10 and C34 stabilize the transition state, and G9 and U37 stabilize the cofactor. Transfection of HEK293T cells with a SAMURI-encoding plasmid enabled the intracellular RNA modification for the subsequent Cu^+^-mediated azide-alkyne cycloaddition (CuAAC) (‘click’) reaction. The ribozyme SAR [109] catalyzes the self-alkylation with an epoxy-activated substrate. In the structure of the ribozyme–substrate complex [110], the pair G17-U42 and the triplet A15-U47-A43 act as key nucleotides that stabilize the substrate in the active conformation.

Silverman’s group produced a DNAzyme that glycosylates the 3′-OH group of DNA using an aryl glycoside substrate in the presence of Zn^2+^ and Mn^2+^. The aryl glycoside derivative was coupled to an additional anchor oligonucleotide for substrate positioning. The same team isolated a DNAzyme for the reductive amination of the substrate’s aldehyde group via the N2 atom of the 5′-terminal guanine, in the presence of Ni^2+^ and NaCNBH_3_ [111]. Later, they created a DNAzyme that catalyzes reductive amination involving the aliphatic amino group of one anchor oligonucleotide and benzaldehyde conjugated to another anchor oligonucleotide [112]. This strategy was then further developed to create DNAzymes for site-specific N-alkylation of nucleobases [113].

#### 2.2.6. Covalent Modification of Peptides

Selection of nucleic acids catalyzing covalent modification of amino acid side chains within peptides expanded the range of techniques for selective protein labeling [114]. For instance, Baskerville and Bartel [115] generated ribozymes that form a stable phosphoamide bond between the 5′ terminus of an RNA and the amino terminus of a specific polypeptide and can be used to supply proteins with amplifiable tags. DNAzymes that catalyze the attachment of RNA to the Tyr side chain via the 5′-phosphate group are described in [116,117,118]. Sachdeva et al. [119] isolated DNAzymes attaching RNA to phosphorylated tyrosine and proposed using them as tools for analyzing protein phosphorylation.

A DNAzyme that catalyzes the reaction between the ε-amino group of Lys and the terminal 5′-phosphate of DNA was generated in [120]. An acylation of the ε-amino group of Lys is also possible by the DNAzyme produced by T. Yao et al. in [121].

As an example of less common peptide-modifying functionality, we should also mention a DNAzyme that introduces an azide group into a peptide for a subsequent CuAAC reaction, using a Tyr-containing hexapeptide and 2′-azido-2′-deoxyadenosine triphosphate as substrates [122].

A series of works by Silverman’s group describes the selection of DNAzymes with tyrosine kinase-like activity [123,124,125]. The DNA library contained a fixed sequence of the ATP aptamer to enhance catalytic efficiency and provide an option for additional regulation. During the selection, the reacted molecules were separated using antibodies against phosphorylated tyrosine.

#### 2.2.7. Carbon–Carbon Bond Formation

**Figure 7:**
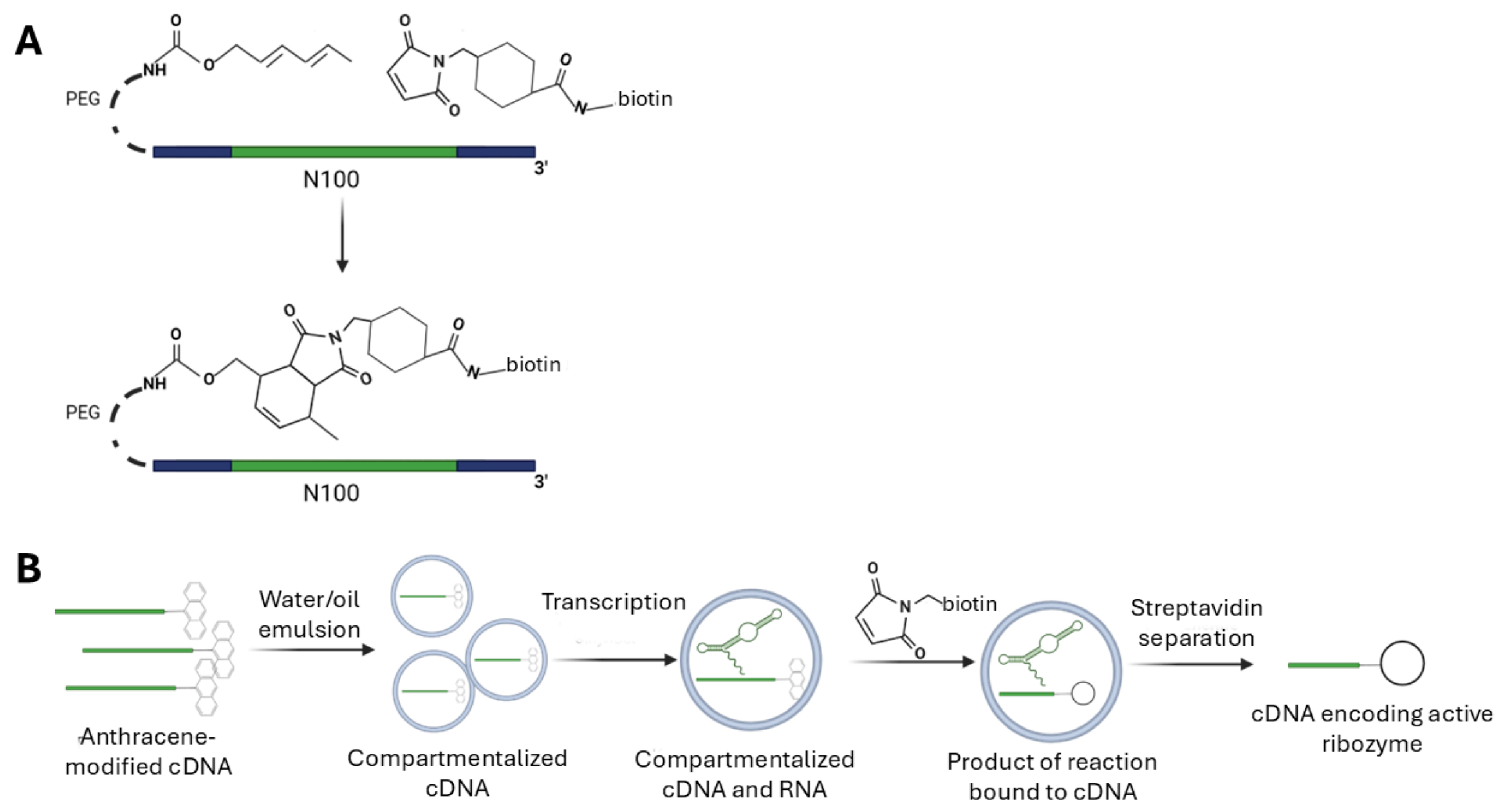
Ribozymes with Diels–Alderase activity. (**A**) selection of Diels–Alderase ribozymes; (**B**) compartmentalization-based selection of ribozymes with Diels–Alderase activity.

The development of ribozyme analogs of Diels–Alderases (enzymes that catalyze Diels-Alder cycloaddition reactions) became an important milestone in the chemistry of catalytic nucleic acids [126,127]. This reaction is essential in organic synthesis and also participates in a number of metabolic pathways [128]. Furthermore, experimental evidence of the ability of ribozymes to catalyze the formation of carbon–carbon bonds is important in the context of the RNA world hypothesis. The RNA library contained a modified uridine analog, 5-pyridylmethylcarboxamide uridine. A diene component was incorporated into the 5′-end of the RNA, and the library was incubated with biotinylated maleimide as a dienophile in the presence of divalent metal cations (Figure 7A). After 12 rounds, the ribozymes acquired the activity that depended on the presence of modified uridine analogs and Cu^2+^ ions. In another study [129], an unmodified RNA library was used for the selection of a ribozyme with Diels–Alderase activity, with anthracene attached to RNA as the diene component and biotin maleimide as the dienophile. The selection and sequence optimization produced a 38 nt ribozyme catalyzing the Diels–Alder reaction in cis (intramolecular reaction). Then, the authors generated a 49 nt Diels–Alderase ribozyme, which performs the in-trans enantioselective cycloaddition of free substrate molecules (not attached to an oligonucleotide) [130]. This ribozyme became one of the most extensively studied artificial catalytic NAs, with an established crystal structure [131], active site architecture, and the mechanism of catalysis investigated using in silico and in vitro methods. The active site represents a pseudoknot with maleimide rigidly fixed within a hydrophobic pocket via stacking, while anthracene is oriented at an angle. Catalysis occurs primarily via spatial complementarity rather than by the acid–base mechanism typical for catalytic NA [132,133,134,135,136,137,138,139]. Interestingly, the ribozyme activity can be allosterically regulated by adding a sequence of theophylline aptamer [140,141]. Later, Silverman’s group demonstrated that DNA can also effectively catalyze the formation of carbon–carbon bonds in the Diels–Alder reaction [142].

Using the example of ribozymes with Diels–Alderase activity, Agresti et al. developed a novel compartmentalization-based selection strategy [143] (Figure 7B). To isolate individual RNAs, they performed the in vitro transcription of the cDNA library with attached anthracene in water-in-oil emulsion droplets, then added Mg^2+^ and biotin maleimide. RNA catalytic activity drove the formation of adducts between single-stranded DNA and the biotinylated reaction product.

#### 2.2.8. Azide-Alkyne Cycloaddition

The DNAzyme CLICK-17 reported in [144] catalyzes one of the most common click chemical reactions, Cu^+^-mediated azide-alkyne cycloaddition (CuAAC). For the selection, the authors employed a ssDNA library supplied with a 5′-alkyne group and an azide-linked biotin for subsequent streptavidin binding and electrophoretic separation. Notably, the Cu^+^ concentration in the selection buffer was lower than that typical for click chemistry. The resulting DNAzyme catalyzes the cycloaddition of various substrates in the presence of either low-concentration Cu^+^ or, alternatively, Cu^2+^ without reducing agents. The last option allows performing click reactions under biocompatible conditions: the authors suggest that CLICK-17 could be used to modify molecules on the cell surface.

#### 2.2.9. Photochemical DNA Repair

**Figure 8:**
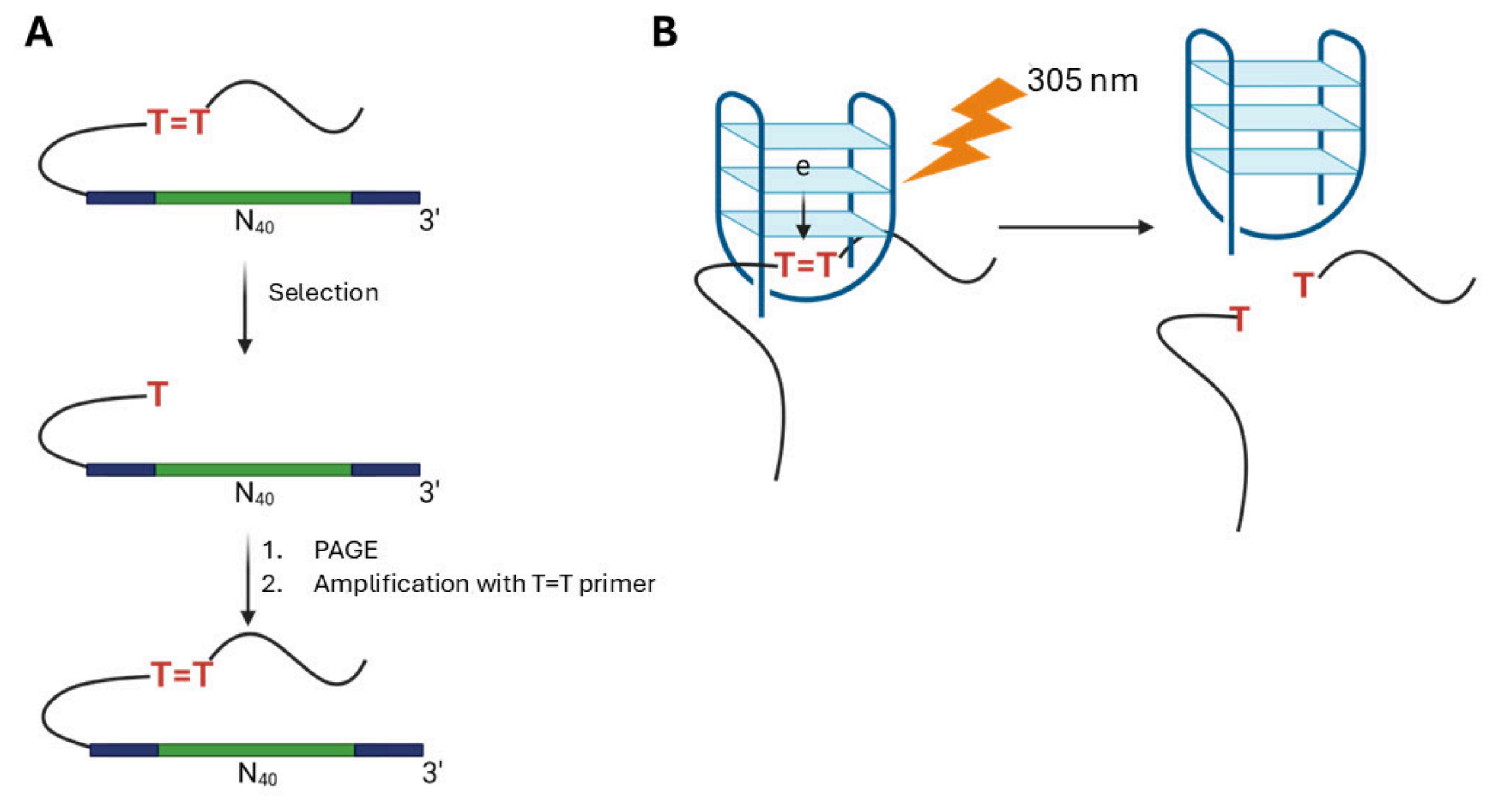
DNAzyme with photolyase activity. (**A**) selection scheme of photolyase DNAzymes; (**B**) proposed mechanism of catalysis.

Chinnapen and Sen [145] created a DNAzyme analog of photolyase, which repairs thymine cyclobutane dimers, a type of DNA damage caused by UV light. The G-quadruplex DNAzyme UV1C catalyzes the UV-dependent reaction without any additional cofactors [146,147] (Figure 8). Another DNAzyme, Sero1C [148], also performs the UV-induced cleavage of TT dimers but requires the presence of serotonin, which suggests the potential for using additional cofactors to enhance the efficiency of DNA catalysis. It was hypothesized that the G-quadruplex maintains the “capture” of near-UV light energy, which facilitates the catalysis.

#### 2.2.10. Peroxidase-like Oxidation

**Figure 9:**
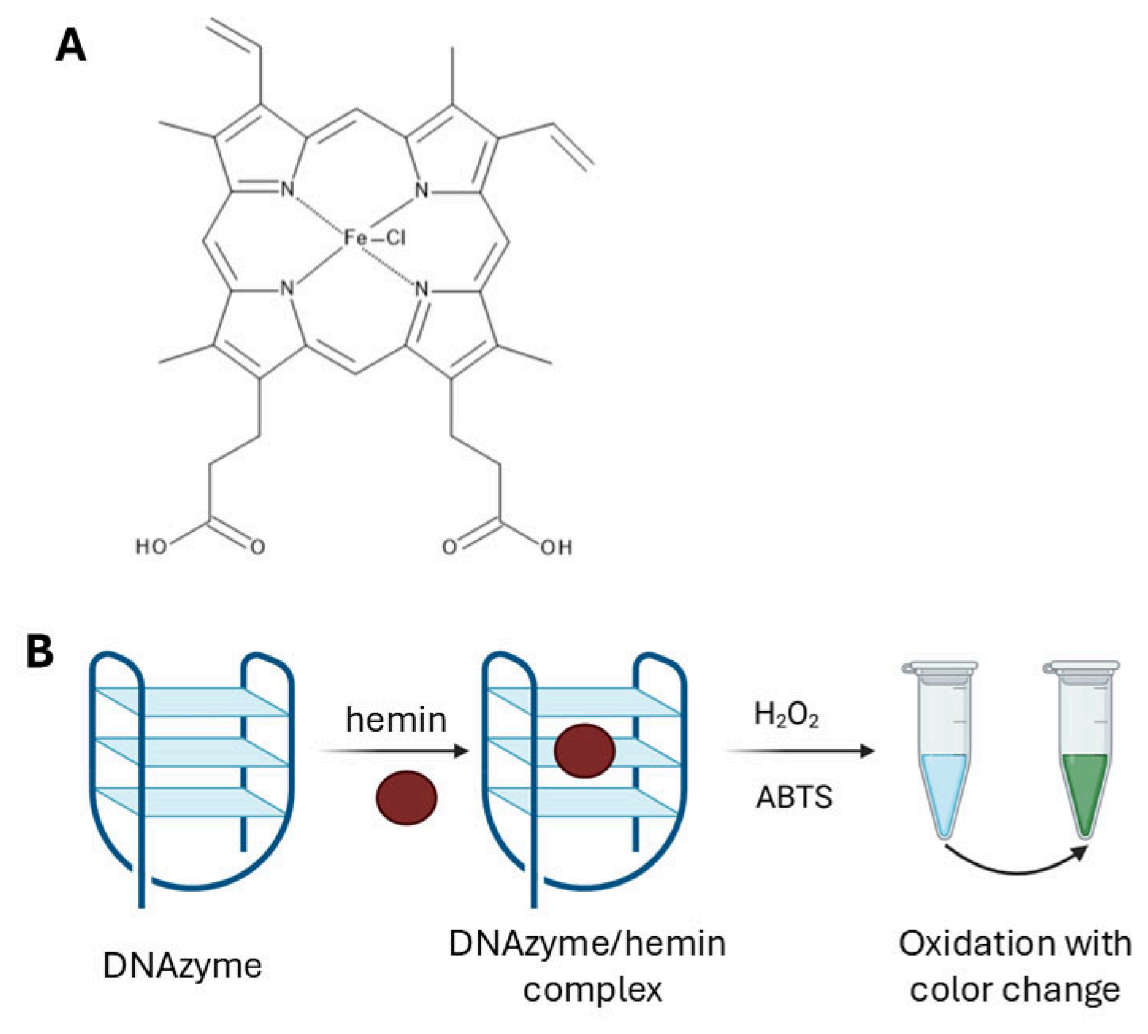
DNAzymes with peroxidase activity. (**A**) hemin complex with Fe^3+^; (**B**) substrate oxidation with peroxidase DNAzyme.

A large set of studies has focused on the production of DNAzymes with peroxidase-like activity, non-protein analogs of the peroxidases widely used in bioanalysis. At the very beginning of this story, Li and Sen generated the PS2.M DNAzyme catalyzing the Cu^2+^ chelation by mesoporphyrin IX. A variation of this G-quadruplex-containing DNAzyme later showed the ability to catalyze the oxidation of organic substrates in the presence of hydrogen peroxide when combined with another porphyrin, Fe^3+^-containing hemin (Figure 9). As in protein peroxidases, substrate oxidation is provided by hemin, which is bound within the G-quadruplex domain of the DNAzyme through stacking interactions [149,150]. The potential for using such DNAzymes as an alternative to horseradish peroxidase to generate a colorimetric signal stimulated the development of a number of other peroxidase-like DNAzymes, containing not only G-quadruplexes but also stem-loop structures as a central element [151,152,153,154,155].

### 2.3. Implications for Prebiotic Chemistry

The discovery of RNA’s catalytic activity stimulated the appearance of the RNA world hypothesis that considers nucleic acids as the first biopolymers capable of both encoding genetic information and catalyzing the set of chemical reactions sufficient for the emergence of the first living organisms. The creation of artificial catalytic NAs performing a variety of hydrolysis and synthesis reactions provided an important piece of evidence supporting this hypothesis [156,157,158].

For instance, Joyce and co-workers used an in vitro evolution of a natural Tetrahymena ribozyme to generate the catalytic RNA that catalyzes the hydrolysis of an amide bond [159]. Later, a series of DNAzymes for hydrolyzing amide and ether bonds was obtained. Silverman’s team selected DNAzymes that hydrolyze ether and aromatic amide bonds [160]. A modified ssDNA library was used for the selection of DNAzymes catalyzing the hydrolysis of aliphatic amides, with thymidine residues replaced by 5′-substituted deoxyuridine (-OH, -COOH, or -NH_2_) [161]. What is most interesting, the unmodified analogs of these DNAzymes retained the ability to hydrolyze aliphatic amide bonds as well.

Several classes of artificial ribozymes demonstrate the potential of RNA to catalyze key biochemical processes: peptidyl transferase ribozymes as “protoribosomes” [162,163]; ribozymes that catalyze the amide bond formation [164]; a ribozyme for the synthesis of uridine monophosphate from ribose phosphate and uracil [165]; and a ribozyme catalyzing a C-S bond formation in a Michael addition reaction [166]. Artificial ribozymes can also catalyze the formation of C–C bonds. Ryu et al. [167] isolated a ribozyme carrying out the Claisen condensation. The selection yielded a set of ribozyme ketosynthases, suggesting that ribozymes may catalyze the synthesis of fatty acids in the ancient RNA world. Fusz et al. [168] created another example of a ribozyme catalyzing the formation of a carbon–carbon bond, namely an aldol condensation.

Cernak and Sen [169] presented an approach for selection of ribozymes utilizing natural cofactors, using the example of a decarboxylation reaction involving thiamine. Additionally, they demonstrated that the ribozyme binds the decarboxylation intermediate product similarly to natural enzymes like pyruvate dehydrogenase.

### 2.4. Bioanalytical Applications of Ribozymes and DNAzymes

The diversity of the available chemical reactions, biocompatibility, and the possibility of using chemical oligonucleotide synthesis make ribozymes and DNAzymes a flexible and convenient basis for creating bioanalytical systems. Self-cleaving ribozymes, RNA-cleaving DNAzymes, and peroxidase DNAzymes serve as recognition or signaling elements in optical and electrochemical biosensors. The range of applications for catalytic nucleic acids is described in detail in comprehensive recent reviews [51,170,171,172,173]. Here, we discuss several representative detection systems that exploit diverse catalytic activities of nucleic acids, thereby illustrating their broad potential for bioanalytical applications.

**Figure 10:**
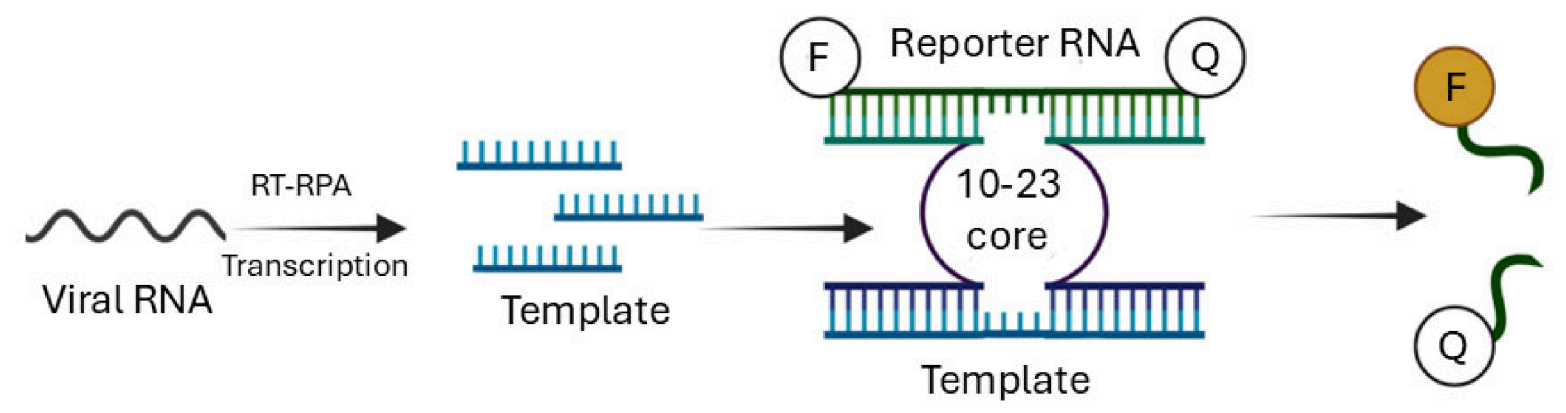
Fluorescent detection of SARS-CoV-2 viral RNA with DNAzyme 10-23 cleaving reporter RNA bearing a fluorophore (F) and a quencher (Q) [174].

RNA-cleaving catalytic nucleic acids can be employed to detect viral RNAs. For example, Yang and Chaput [174] designed a system based on the 10-23 DNAzyme for detecting SARS-CoV-2 viral RNA in patient-derived samples (Figure 10). After reverse transcription with recombinase-mediated polymerase amplification of the sample and T7 transcription, the product becomes a template for the assembly of a DNAzyme complex that cleaves the reporter RNA bearing a fluorophore and a quencher, giving a fluorescent signal. The detection limit of the platform is as low as 10 copies/µL, with an assay time of 1 h.

**Figure 11:**
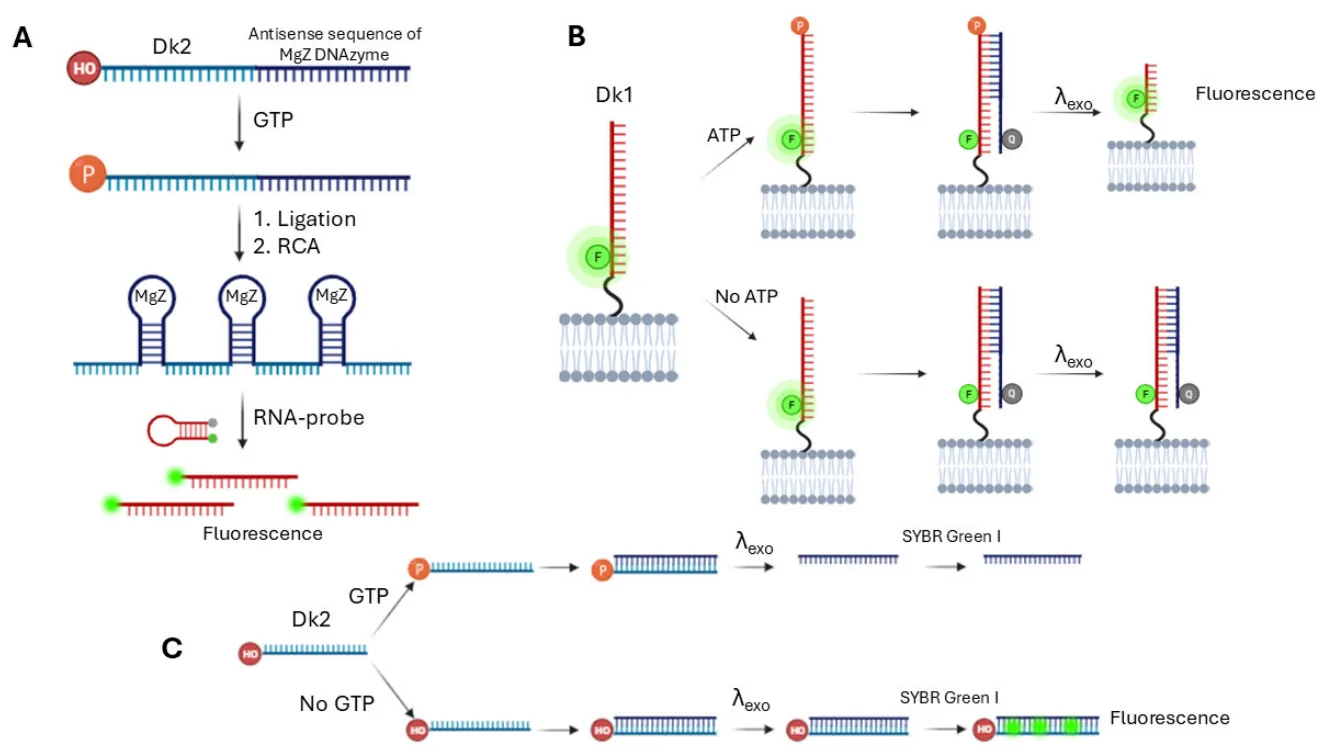
Detection of GTP and ATP with DNAzymes. (**A**) GTP detection system with DNAzymes Dk2 and MgZ: in the presence of GTP, the 5′-end of Dk2 is phosphorylated, allowing the molecule to be ligated into a ring structure for subsequent RCA amplification; the RCA product contains the sequence of the MgZ DNAzyme, which cleaves the RNA probe to activate fluorescence [175]; (**B**) detection of extracellular ATP: DNAzyme Dk1 bearing a fluorophore (F) is immobilized on the cell surface via cholesterol residue; in the presence of ATP, phosphorylation of the 5′-end occurs; following the addition of a partially complementary strand with a quencher (Q), the fluorescence intensity decreases; the λ exonuclease cleaves the phosphorylated strand, and the fluorescence intensity increases again [179]; (**C**) GTP detection with SYBR Green I: in the presence of GTP, the 5′-end of Dk2 is phosphorylated; following the addition of a complementary oligonucleotide and λ exonuclease treatment, the phosphorylated strand is cleaved, so SYBR Green I addition does not induce a fluorescent signal [178].

DNAzymes with polynucleotide kinase-like activity became a basis for fluorescence-based GTP detection systems. The platform relies on signal amplification via RCA and an RNA-cleaving DNAzyme [175,176] or an additional fluorescent probe [177,178]. The same approach was employed as well for the detection of extracellular ATP [179] (Figure 11).

**Figure 12:**
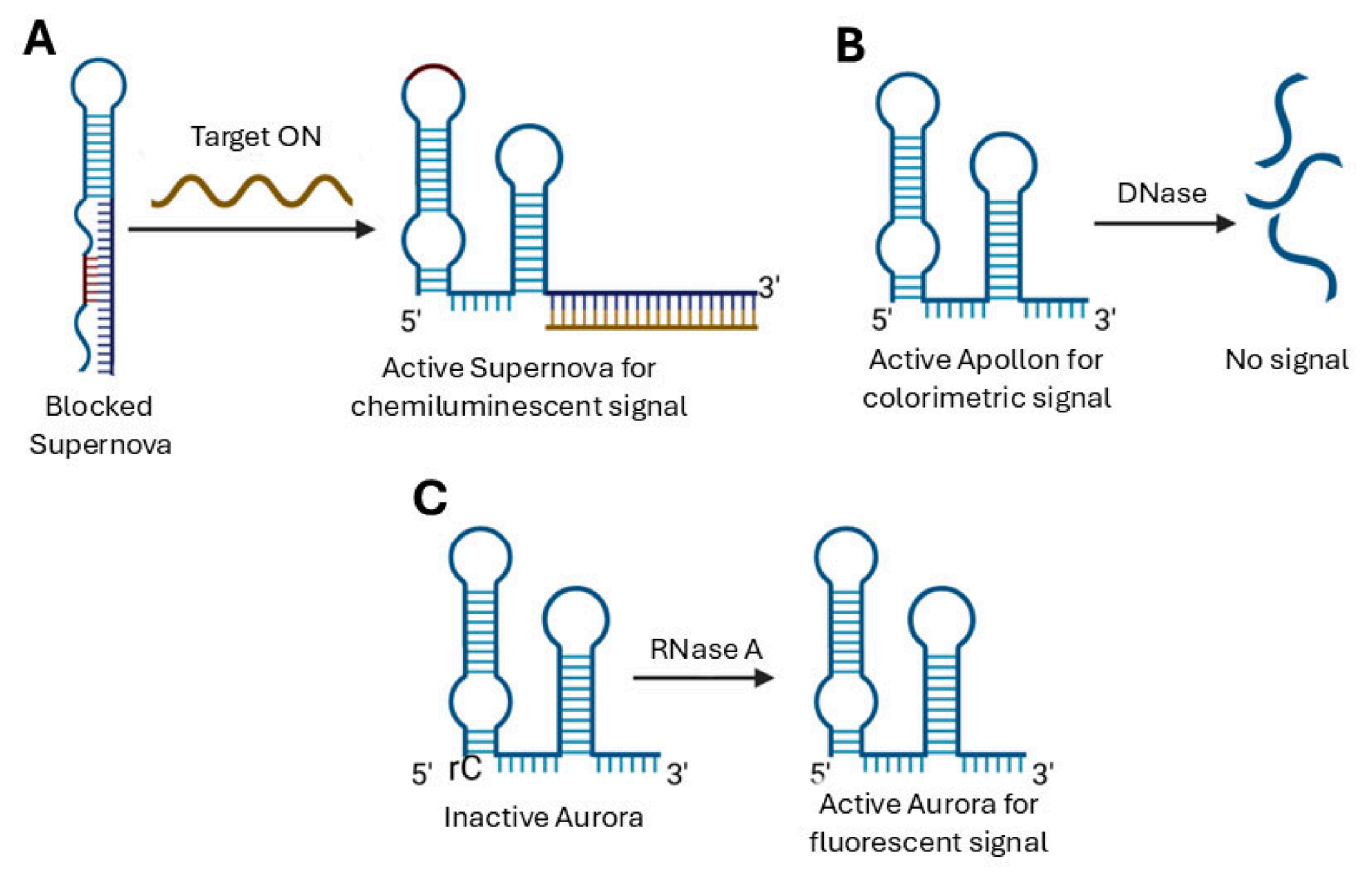
Probes based on DNAzymes of the Supernova family. (**A**) oligonucleotide detection with Supernova; (**B**) DNase I detection with Apollon; (**C**) RNase A detection with Aurora.

Phosphatase DNAzymes of the Supernova family found their application for the detection of different molecules. Namely, Supernova DNAzyme can be adapted to identify DNA fragments of specific sequences (Figure 12A) [96]. Probes based on Apollon DNAzyme allowed detecting RNase A and DNase I (Figure 12B) in solution [98], and Aurora-based probes were developed to screen for inhibitors of the Nsp15 ribonuclease and to detect RNase A (Figure 12C) [99].

**Figure 13:**
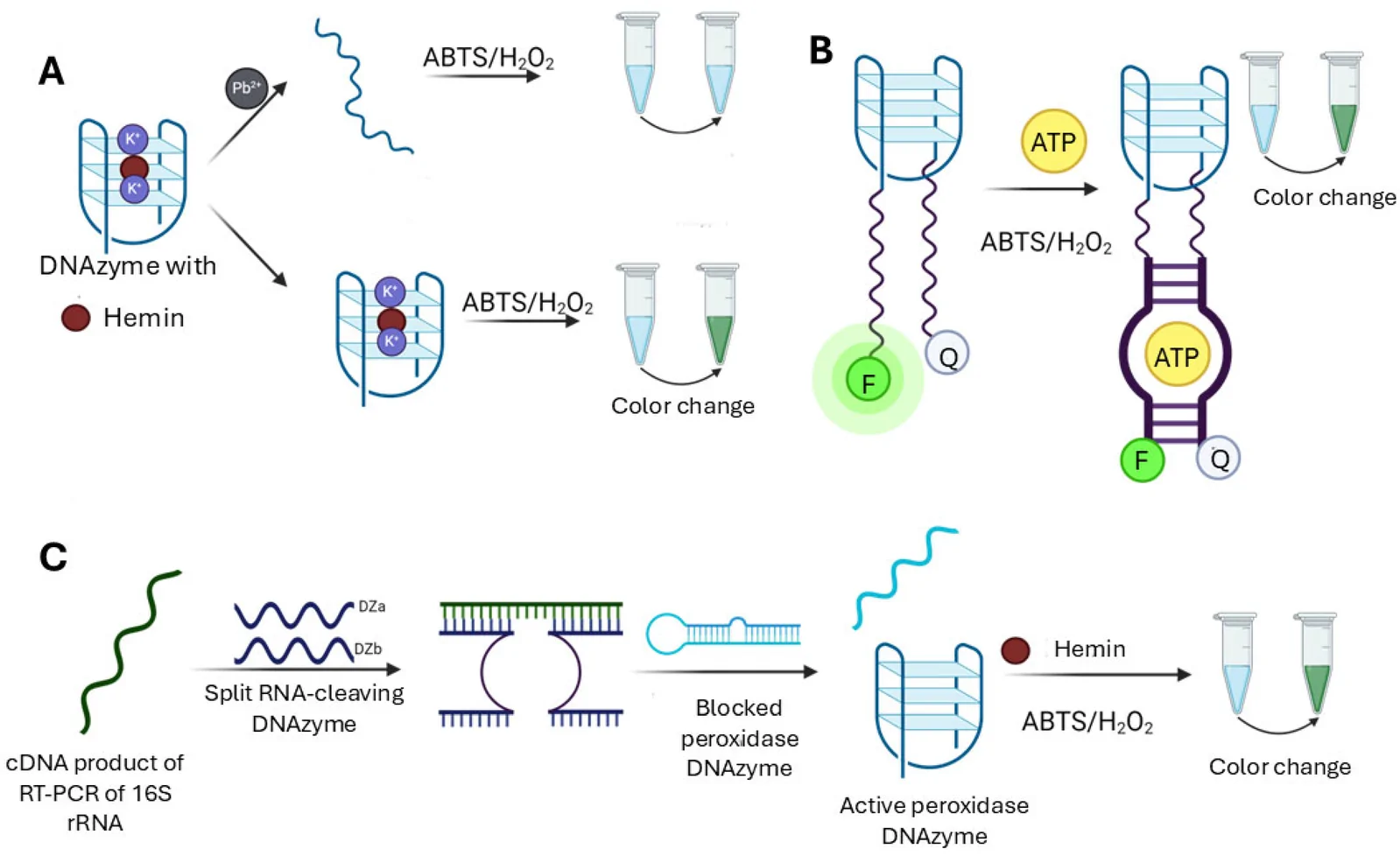
Detection systems for various analytes based on peroxidase-like DNAzyme. (**A**) Pb^2+^ detection system [189]. The quadruplex is stable in the absence of Pb^2+^; the addition of the substrate ABTS (colorimetric analysis) or luminol (chemiluminescent analysis) and subsequent oxidation produce a signal. Pb^2+^ ions destabilize the complex with the loss of DNAzyme activity and signal disappearance; (**B**) ATP detection system [190]. The DNAzyme core is flanked by two aptamer fragments bearing a fluorophore (F) and a quencher (Q). In the presence of ATP, they form an aptameric structure that favors fluorescence quenching. At the same time, the peroxidase-like activity of DNAzyme increases in the presence of ATP [192], providing a color change upon addition of a chromogenic substrate; (**C**) mycobacteria detection system [191]. The RNA-hydrolyzing DNAzyme is split in two chains. In the presence of species-specific cDNA produced after RT-PCR of 16S rRNA, they form an active complex that cleaves the blocking RNA from the peroxidase DNAzyme. Free DNAzyme in the presence of K^+^ and hemin oxidizes the ABTS substrate to produce a colored solution.

The peroxidase-like activity of hemin-dependent DNAzymes allows using them in detection systems with an electrochemical or optical signal (colorimetric, luminescent, or fluorescent) [180,181]. Thanks to the chemical nature of oligonucleotides, DNAzymes combine well with nanomaterials or isothermal amplification techniques to increase analytical signal [6,182]. Their activity can be regulated with complementary oligonucleotides [183,184,185,186] or additional cofactors [187]. Moreover, systems based on peroxidase DNAzymes have an option of regulation by other functional nucleic acids, such as RNA-cleaving DNAzymes [6] or aptamers [185,188]. T. Li et al. proposed a method for detecting Pb^2+^ ions based on their ability to denature the DNAzyme active structure [189] (Figure 13A). Kang et al. [190] combined a DNAzyme and an ATP-binding aptamer to create an ATP sensor (Figure 13B). H.N. Wood et al. [191] designed a system for the detection of clinically significant non-tuberculous mycobacteria by their 16S rRNA, utilizing a combination of RNA-cleaving and peroxidase-like DNAzymes (Figure 13C).

**Figure 14:**
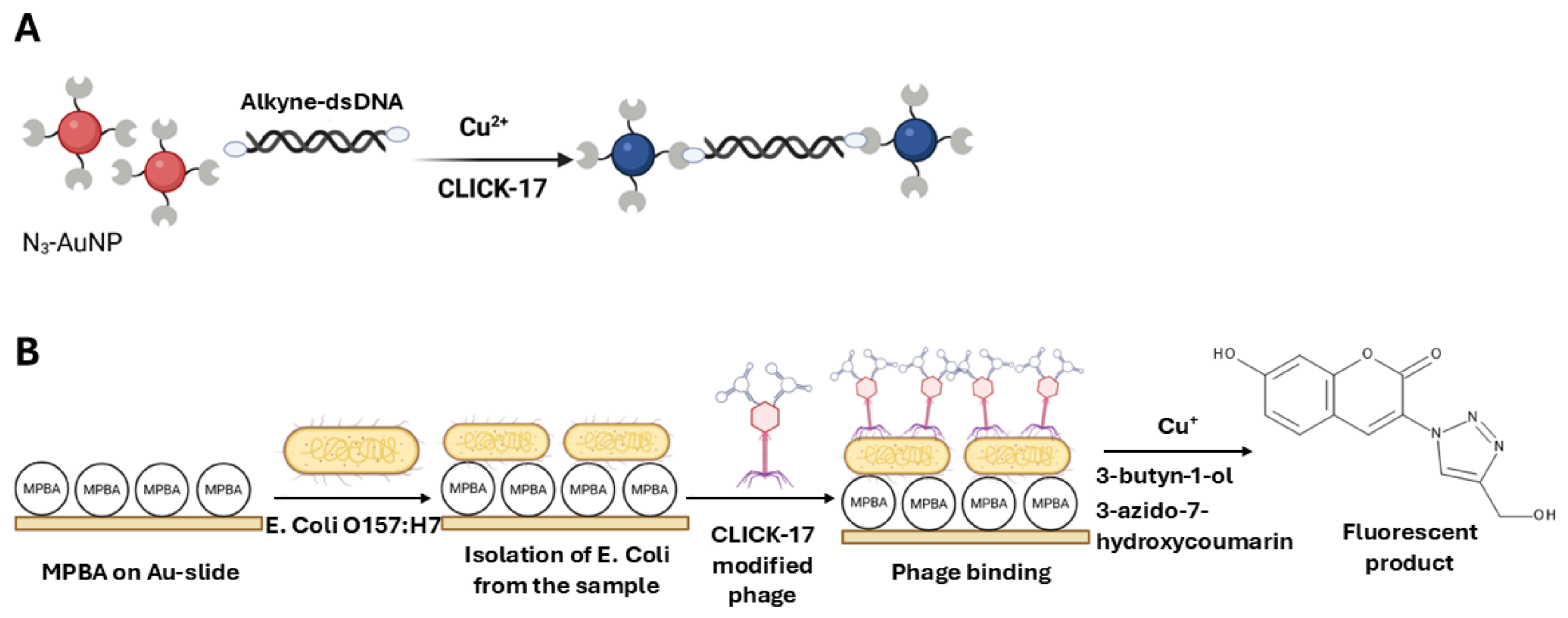
Detection systems with CLICK-17 DNAzyme. (**A**) Cu^2+^ detection with gold nanoparticles [194]. The click reaction provides an aggregation of gold nanoparticles with a red to blue color shift; (**B**) *E. coli* detection with CLICK-17-modified phage and click-dependent formation of the fluorescent product [197].

DNAzyme CLICK-17 applications include immobilizing ethynylferrocene on an electrode surface for the development of electrochemical sensors [193] and facilitating a click reaction in the optical detection system for Cu^2+^ ions using gold nanoparticles [194] (Figure 14A). An alternative system for detecting copper ions was developed using the truncated version, CLICK-T [195]. The CLICK-17 DNAzyme also acted as a part of fluorescent probes for the detection of bacterial pathogens: the DNAzyme, conjugated to a recognition element (an antimicrobial peptide or a phage), catalyzed the formation of a fluorescent product (Figure 14B) [196,197].

The bioanalytical application of catalytic NAs covers a wide range of targets, involves the use of readily available equipment, and enables achieving low detection limits in model solutions (Table 3). However, it is rather difficult to unequivocally assess the practical performance of such bioanalytical systems based on the characteristics reported in the original studies. The assays often require multi-step pretreatment or target purification, and their sensitivity often has not been investigated in real samples.

## 3. Fluorescent Light-Up Aptamers Binding Small-Molecule Fluorogens

Fluorescent light-up aptamers represent a distinctive class of aptamers that can be considered as close analogs of catalytic nucleic acids. They bind a certain small-molecule fluorogen and stabilize it in a conformation most optimal for fluorescence excitation and emission. This type of aptamers attracts particular interest due to their great bioanalytical potential.

An RNA sequence corresponding to a “light-up” aptamer motif can be expressed in cells as a part of the mRNA of a gene of interest and then provide fluorescent bioimaging upon the introduction of an appropriate fluorogen. Therefore, the creation of aptamer-fluorogen pairs focuses on the fluorescent properties of the system, considering the long-wavelength red part of the spectrum as the optimal range for excitation and emission [198]. The radiation in this range lies in the tissue transparency window, which allows researchers to apply these technologies both at the cellular level and in small laboratory animals. The ability of the low-molecular-weight fluorogen to enter cells, along with its low intrinsic toxicity, is also an important criterion. [199]. A fairly wide variety of light-up systems has now been developed. Interestingly, light-up aptamers are usually named after plants: vegetables, fruit, or flowers.

**Figure 15:**
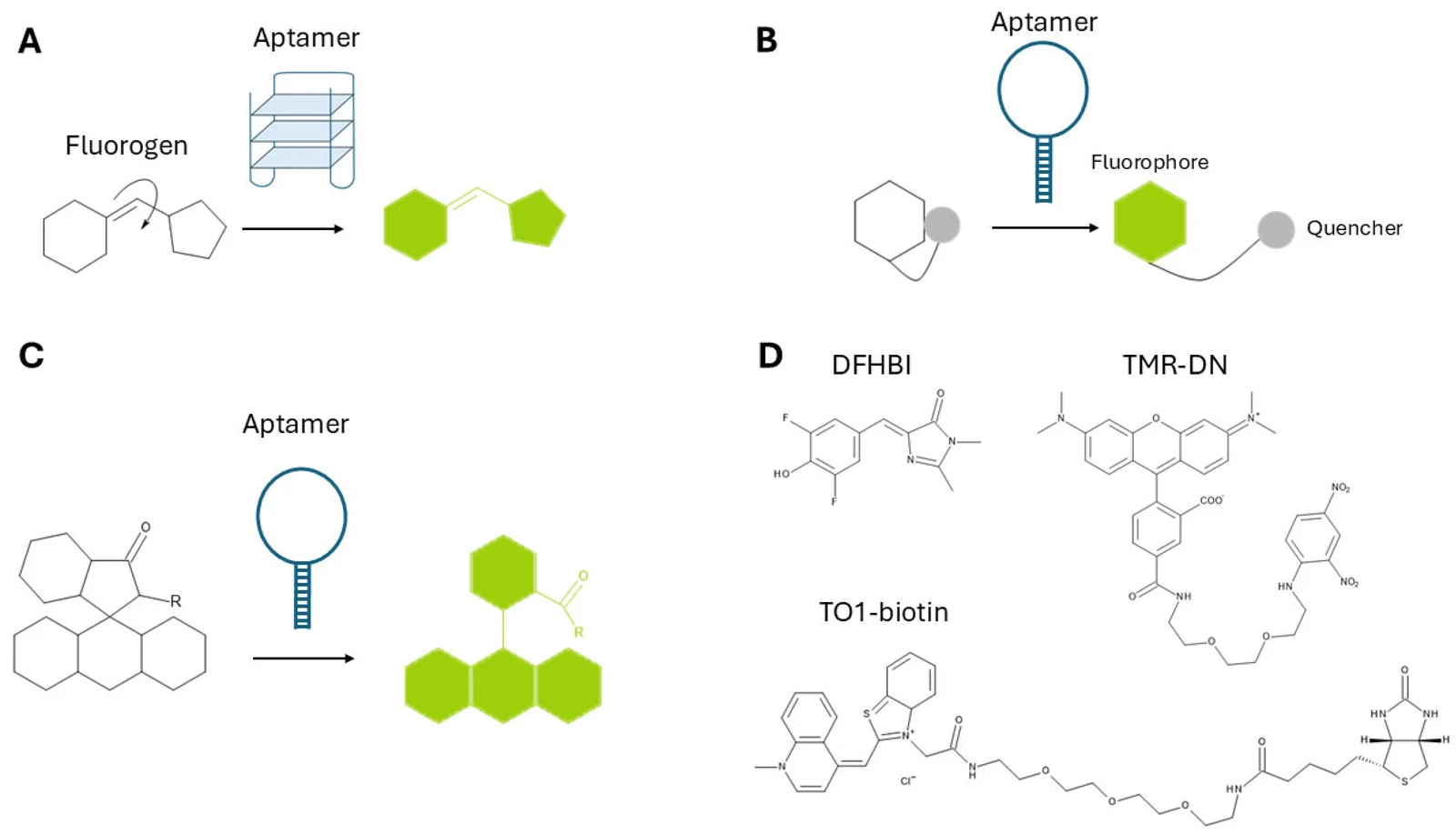
Light-up aptamers fluorogenic mechanisms. (**A**) immobilization/planarization of the fluorogen by the aptamer; (**B**) fluorophore–quencher separation; (**C**) binding-induced ring opening; (**D**) examples of fluorogens for light-up aptamers.

As a rule, aptamers of this type contain the G-quadruplex as an essential structural element. In its free form, the fluorogen molecule exhibits no or weak fluorescence due to the existence of several alternative conformations. Binding to the aptamer fixes the fluorogen into its optimal conformation for emission, thereby suppressing non-radiative energy dissipation, which greatly enhances the efficiency of fluorescence (Figure 15A) [7,8,199,200,201].

The production of light-up aptamers also relies on the SELEX methodology. An RNA aptamer for the dye malachite green (MGA) became the first example of such a light-up system. However, the cytotoxicity of MGA significantly limited its use [202,203]. The Spinach-DFHBI (Figure 15D) system [204] and its later variants—iSpinach [205], Spinach2 [206], and Baby Spinach [207]—employed a fluorogen analogous to HBI (the GFP fluorophore), which made them cell-compatible. Subsequently, the selection of light-up aptamers was further improved by combining SELEX with fluorescence-activated cell sorting (FACS). After initial enrichment of the library by selection for fluorogen, the RNA pool was expressed in cells, most commonly *E. coli*, followed by the isolation of only those cells that “light-up” in the presence of the fluorogen. The approach thus enabled the direct selection of biocompatible sequences. This technology, for example, produced the Broccoli-DFHBI-1T light-up pair [7,199]. Aptamer/fluorogen systems with fluorescence in the longer-wavelength red region include the Corn-DFHO [208], Mango I-TO1-Biotin (Figure 15D) [209], Pepper-HBC without G-quadruplex elements [210], and other RNA aptamers [7,203,211]. A whole variety of them is summarized in Supplementary Materials, Table S1.

The examples of light-up DNA aptamers are few in number and include aptamers to DFHBI-1T [212], TO1-biotin (Figure 15D) [213], crystal violet [214], and some other fluorogens (Table S1). Light-up DNA aptamers are primarily considered as components of in vitro detection systems, as their main advantage is greater stability compared with RNAs.

An alternative approach to the design of aptamer-based light-up systems employs the fluorogens that contain both a fluorophore and a quencher in their structure. Binding to the aptamer locks their configuration in a position where the quencher is distant from the fluorophore (Figure 15B) [200,203]. This approach has been implemented, for example, in the RhoBAST light-up aptamer (Figure 15D) [215].

**Figure 16:**
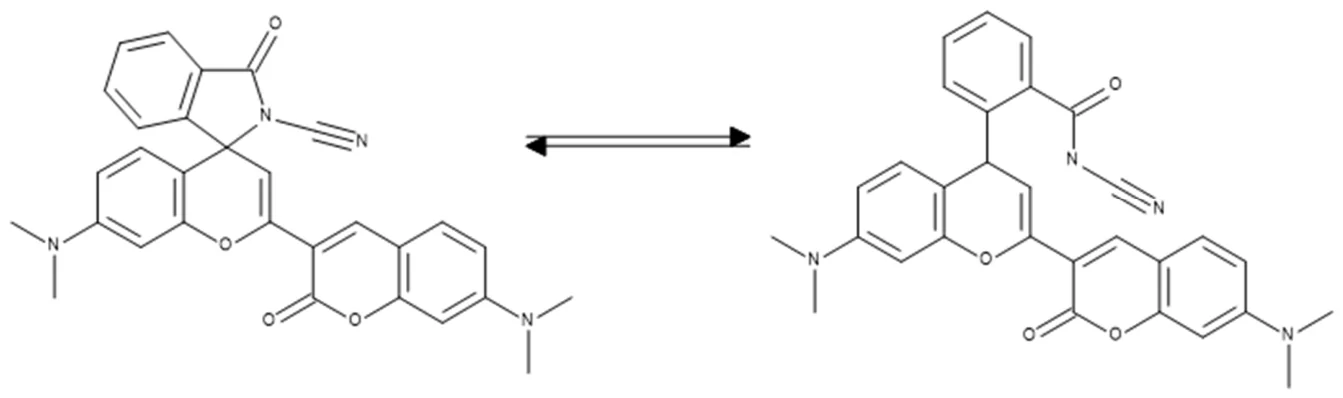
Transition between forms of fluorophore in light-up pair BeCA-BC6.

The next generation of light-up RNAs relies on the use of a spirocyclic fluorophore that undergoes ring-opening after binding to the aptamer (Figure 15C) [200]. For example, the BeCA-BC6 pair [216] uses the BC hybrid fluorophore based on benzopyrylium and coumarin. The RNA aptamer induces a fluorescence change during the transition from a spirolactam (fluorescence emission in the blue region of the spectrum) to a zwitterion (fluorescence emission in the far-red region). This approach allowed for simultaneous monitoring of both free and RNA-bound fluorophores within the cell and provided more reliable data on the quantity and localization of the RNA of interest. Interestingly, both the nucleic acid and the fluorophore evolved within the framework of the proposed methodology. During the selection, the researchers used a variant of the fluorophore that in aqueous solutions at physiological pH exists predominantly as a zwitterion. After the identification of the aptamer, the dye structure was further modified to achieve an optimal ratio of the cyclic and open forms (Figure 16). The resulting BC6 fluorophore, when paired with the BeCA aptamer, visualized the distribution of free BC6 and aptamer-containing RNA within cells.

Aptamer/fluorogen light-up systems offer a high signal-to-noise ratio; most of them are biocompatible. The most representative examples are given in the Table 4. Furthermore, one can combine them with other aptamers, split them into sequence fragments for more precise activity regulation, or amplify them using RCA, CHA, or LAMP methods [199,203,211,217,218,219].

The use of aptamer/fluorogen pairs for analytical purposes is becoming increasingly widespread. Such systems are used to label RNA molecules [7]; as reporters in biosensors [227] for the detection of small molecules [228], nucleic acids, and proteins; and for the in vivo visualization of RNA and metabolites in living cells [198,200,229,230,231,232,233,234,235,236], among other applications. For instance, the Squash-DFQL-1T pair provides a photostable signal in the NIR region of the spectrum, thus enabling the visualization of ncRNA and microRNA-21 in cells and even in mice [226]. Wang et al. [237] developed an approach to the in situ visualization of mRNA based on the Broccoli-DFHBI split system; the method does not require any modifications to the mRNA molecule.

## 4. Conclusions and Future Directions

An analysis of the entire body of published work on the development of catalytic nucleic acids and fluorescent aptamers shows that attention is focused primarily on expanding both the spectrum of catalyzed reactions and the variety of aptamer/fluorogen combinations, as well as their analytical applications. In contrast, a much lower proportion of studies concentrates on the structure that maintains the catalytic function or favors fluorogen activation.

While natural ribozymes have been rather thoroughly investigated in terms of catalytic mechanisms, artificially selected catalytic DNAs and RNAs still remain less studied. The catalytic activity of many classes of artificial ribozymes and DNAzymes strictly depends on the presence of metal ions; however, their precise role has been clearly proved only for a relatively small group. In the case of acid–base catalysis by RNA-cleaving DNazymes, hydrated metal ions stabilize the leaving group, whereas in the case of the 9DB1 DNAzyme, they function as electrostatic organizers. For alkylating ribozymes and the Diels-Alder ribozyme, metal cations provide structural support for the active site. The nucleobases of the active site act as general bases and form stacking structures that ensure the precise positioning of the substrate and cofactors.

For nucleolytic ribozymes and DNAzymes, with well-understood catalytic cores, the hybridization “arms” flanking the core allow for adaptation to a specific ON target, an approach widely used in the development of sensors and therapeutic systems. However, the lack of data on the mechanisms of catalytic activity of other classes of artificial catalytic NAs, despite the wide range of available reactions, limits their versatility for practical applications.

Meanwhile, as experimental in vitro selection is often challenging to control and guide, the rational design of functional NAs (in silico selection and in silico maturation) is gaining popularity. This strategy leverages molecular modeling, docking techniques, and machine learning methods [238,239]. For instance, to improve the performance of a peroxidase-like quadruplex DNAzyme, Fenati et al. [240] staryed from a previously reported structure and combined molecular docking with experimental evaluation of catalytic activity. To date, a wide range of software has been developed for modeling the spatial structures of functional NAs and their complexes with ligands (see, e.g., the reviews [241,242]). At the same time, the need for further improvement of existing algorithms is always noted, in particular, for an adequate description of the structure of modified nucleotides.

Artificial intelligence can also accelerate other steps of catalytic NA engineering. Machine-learning models can analyze sequence/structure relationships within datasets from high-throughput sequencing after SELEX and rank candidates according to predicted catalytic activity or substrate binding affinity and compatibility with certain reaction conditions [243,244,245]. Generative models can explore the regions of sequence space that are poorly represented in conventional libraries and generate new catalytic NAs and aptamers [244,246]. In the longer term, active-learning cycles that iteratively connect model predictions with chemical synthesis and biochemical assessment may reduce the number of wet selection rounds and enable multi-parameter optimization of activity, selectivity, stability, and cellular performance.

A prerequisite for employing AI- and ML-driven strategies is the most comprehensive possible dataset on the structures of active sites and substrate complexes for already known ribozymes, DNAzymes, and light-up aptamers. Therefore, we can confidently anticipate that in the coming years, the key focus areas (“hot spots”) in this field will shift to the studies of three-dimensional structures and the completion of “structure–function” databases, as well as to the further refinement of molecular modeling approaches and in silico selection of functional NAs. The rapid advancement of structural research methods, coupled with the explosive growth in the number and variety of computational techniques, holds promise for the development of robust and versatile platforms for designing ribozymes, DNAzymes, and aptamer-based fluorophores with a desired set of functions to address a wide range of biotechnological and bioanalytical challenges.

## Figures and Tables

**Table 1:** Proposed catalytic mechanisms for artificial ribozymes and DNAzymes.

Reaction Class	Representative	Nucleobases Role	Cofactor Role	Structural Evidence
Phosphodiester bond cleavage	8-17	G13 acts as a general base and deprotonates the 2′-OH of the substrate	Hydrated metal ions (Pb^2+^, Zn^2+^, Mg^2+^) act as a general acid and stabilize the leaving group	X-ray, cryo-EM, NMR
	10-23	G14 acts as a general base and deprotonates 2′-OH of the substrate		
Phosphodiester bond formation	9DB1	Organization of the reactive complex; dA13 forces 3′-5′ regioselectivity	Na^+^ and Mg^2+^ ions bridge and orient reactive atoms to overcome charge repulsion	X-ray
Phosphoryl-transfer	Supernova	Proposed mechanism includes G(A/G) motifs for Zn^2+^ coordination	Proposed mechanism includes two catalytic Zn^2+^ in the active site	Not structurally resolved
Alkylation	MTR1	C10 and U45 align O6-methylguanine for nucleophilic attack by A63; C10 acts as a general base to protonate the resulting guanine	The exogenous O6-methylguanine acts as a methyl donor	X-ray
	SMRZ-1	G27 provides nucleophilic attack from its N7 atom; G29 coordinates Cu^2+^; G12, C25, A10 provide stacking platform and positioning near target G27	SAM serves as a methyl donor; Cu^2+^ coordinates to the carboxyl and amino groups of SAM and the O6/N7 atoms of G29	X-ray
	SAMURI	Four stacked layers of the active core strictly arrange ProSeDMA and expose nucleophilic A52	ProSeDMA serves as an alkyl donor; Mg^2+^ stabilizes the fold	X-ray
Diels-Alder reaction	49mer	G9, U17 via two hydrogen bonds activate dienophile, G1-G4, G24, G2–C10–U17 form strict active site fold for substrate positioning	Mg^2+^ supports the active site architecture	X-ray, NMR
Peroxidase-like oxidation	PS2.M	DNAzyme forms G4-quadruplex structure for hemin binding and catalysis enhancement	Hemin provides the Ox-Red reaction; K^+^ stabilizes the G4-quadruplex	Not structurally resolved

**Table 3:** Representative examples of bioanalytical systems based on catalytic nucleic acids.

Target	Recognition and Catalytic Element	Matrix	Pre-Amplification	Type of Signal	Reported LOD, Assay Time
SARS-CoV-2 viral RNA	Split X10-23 DNAzyme	Viral RNA extracted with commercial kit	RT-RPA, T7 Transcription	Fluorescence	10 copies/μL, 1 h
GTP	Dk2 DNAzyme, MgZ RNA-cleaving DNAzyme	Buffer	RCA	Fluorescence	25 nM
	Dk2 DNAzyme	Spiked serum		Fluorescence	340 nM
ATP	Cholesterol modified Dk1 DNAzyme	Live cell extracellular environment		Fluorescence	10 μM
Model ON	Supernova DNAzyme	Buffer		Chemiluminescent	NA
DNase I	Apollon DNAzyme	Buffer		Colorimetric	10–100 nM
RNase A	Aurora DNAzyme	Buffer		Fluorescence	100 pM
Mycobacterial 16S rRNA	RNA-cleaving DNAzyme, Peroxidase DNAzyme		RT-PCR	Colorimetric	4900 genome equivalents
*E. coli* O157:H7	CLICK-17 conjugated to a phage			Fluorescence	50 CFU/mL, 30 min

**Table 4:** Representative examples of fluorescent light-up aptamers.

Aptamer	Fluorogen	Activation Mechanism	Aptamer Structural Motif	λ_ex_/λ_em_, nm	Brightness	Turn-On	Cellular/In Vivo Application Examples
Corn	DFHO	Fluorogen immobilization	G-quadruplex	505/545	7250	417	[208]
Spinach	DFHBI	Fluorogen immobilization	G-quadruplex	469/501	17,500	2000	[220,221]
Broccoli	DFHBI-1T	Fluorogen immobilization	G-quadruplex	472/507	27,800	800	[220,222]
	BI			470/505	22,500	1100	
Mango I	TO1-Biotin	Fluorogen immobilization	G-quadruplex	510/535	11,000	1300	[223,224]
Pepper	HBC-derivatives	Fluorogen immobilization	non-G-quadruplex tuning-fork-like architecture	577/620 (HBC620)	43,098	12,600	[225]
Squash	DFHO	Fluorogen immobilization	fork-like architecture formed by three A-form double helices	505/545	14,760	550	[226]
RhoBast	TMR-DN	Fluorophore-quencher separation	a four-way junction	564/590	55,000	26	[215]
BeCA	BC6	Binding-induced ring opening	Hairpin structure	420/478, 665/684	NA	15	[216]

## Data Availability

Data sharing is not applicable to this article.

## Supplementary Materials

The following supporting information can be downloaded at https://www.mdpi.com/article/10.3390/molecules31213861/s1, Table S1: Fluorescent light-up aptamers, fluorogen structures and characteristics [247,248,249,250,251,252,253,254,255,256,257,258,259,260,261,262].

## Abbreviations

The following abbreviations are used in this manuscript:

4-MUP	4-methylumbelliferyl phosphate
ABTS	2,2′-azinobis(3-ethylbenzothiazoline-6-sulfonic acid)
AI	Artificial intelligence
AuNP	Gold nanoparticles
CHA	Catalytic hairpin assembly
CuAAC	Cu^+^-mediated azide-alkyne cycloaddition
DFHBI	(Z)-4-(3,5-difluoro-4-hydroxybenzylidene)-1,2-dimethyl-1H-imidazol-5(4H)-one
DFHBI-1T	(Z)-4-(3,5-difluoro-4-hydroxybenzylidene)-2-methyl-1-(2,2,2-trifluoroethyl)-1H-imidazol-5(4H)-one
DFHO	3,5-difluoro-4-hydroxybenzylidene imidazolinone-2-oxime
DFQL-1T	(5Z)-5-[(3,5-difluoro-4-hydroxyphenyl)methylene]-3,5-dihydro-2-methyl-3-(2,2,2-trifluoroethyl)-4H-imidazol-4-one
FACS	Fluorescence-activated cell sorting
FIA	Fluorescein iodoacetamide
GFP	Green fluorescent protein
glmS	Glucosamine-6-phosphate dependent ribozyme
HBC	4-[(Z)-1-cyano-2-[4-[2-hydroxyethyl (methyl) amino]phenyl]vinyl] benzonitrile
HBI	4-hydroxybenzylidene imidazolinone
Hcr	Hatchet ribozyme
HDVr	Hepatitis delta virus ribozyme
Hhr	Hammerhead ribozyme
Hpr	Hairpin ribozyme
LAMP	Loop-mediated isothermal amplification
MGA	Malachite green aptamer
MPBA	4-mercaptophenylboronic acid
ML	Machine learning
NA	Nucleic acids
ncRNA	Non-coding RNA
NIR	Near-infrared
PAGE	Polyacrylamide gel electrophoresis
Plr	Pistol ribozyme
pNPP	p-nitrophenyl phosphate
ProSeDMA	Propargylated Se-2,6-diaminopurine-selenomethioninamide
RCA	Rolling Circle Amplification
RT-RPA	Reverse transcription with recombinase-mediated polymerase amplification
SAM	S-adenosylmethionine
SARS-CoV-2	Severe acute respiratory syndrome-related coronavirus 2
SELEX	Systematic Evolution of Ligands by Exponential Enrichment
TMR-DN	tetramethylrhodamine-dinitroaniline
TO1	Thiazole orange 1
Twr	Twister ribozyme
Twsr	Twister sister ribozyme
VSr	Varkud satellite ribozyme
