# Supplementary material for: Ribozymes, DNAzymes, and Fluorescent Aptamers: Diversity, Mechanisms, and Prospects for Bioanalytical Applications
Source: Molecules. 2026 Oct 26;31(21):3861. doi: 10.3390/molecules31213861 (PMC13764992; doi:10.3390/molecules31213861)
Supplement: Supplementary file 1 [file molecules-31-03861-s001.zip › molecules-4597800-supplementary.pdf]

Table S1. Fluorescent light-up aptamers, fluorogen structures and characteristics

Aptamer	Sequence, 5'-3'	Fluorogen		$\lambda_{ex}/\lambda_{em}$, nm	Ref.
RNA aptamers					
MGA	GGAUCCCGACUGGCGAGAGCCAGGUAACGAAUGGAUCC	MG (malachite green, 4-[4-{[4-(Dimethylamino)phenyl](phenyl)methylidene}-N,N-dimethylcyclohexa-2,5-dien-1-iminium chloride)	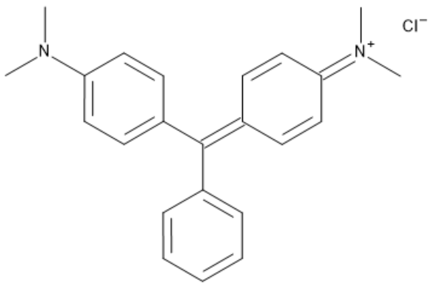	630/650	[202]
Spinach	GACGCAACUGAAUGAAAUGGUGAAGGACGGGUCCAGGUGUGGCUGCUUCGGCAGUGCAGCUUGUUGAGUAGAGUGUGAGCUCCGUAACUAGUCGCGUC	DFHBI ((Z)-4-(3,5-difluoro-4-hydroxybenzylidene)-1,2-dimethyl-1H-imidazol-5(4H)-one)	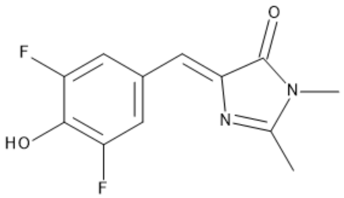	469/501	[204]
Broccoli	GAGACGGUCGGGUCCAGAUAUUCGUAUCUGUCGAGUAGAGUGUGGGCUC	DFHBI-1T ((Z)-4-(3,5-difluoro-4-hydroxybenzylidene)-2-methyl-1-(2,2,2-trifluoroethyl)-1H-imidazol-5(4H)-one)	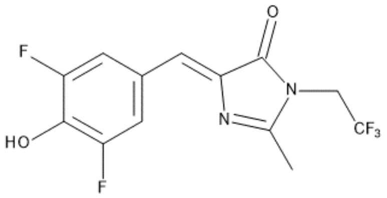	472/507	[247]

		BI (((Z)-3-((1H-benzo[d]imidazol-4-yl)methyl)-5-(3,5-difluoro-4-hydroxybenzylidene)-2-methyl-3,5-dihydro-4H-imidazol-4-one)	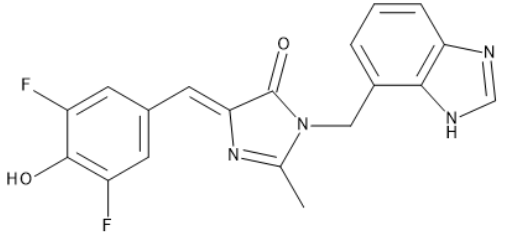	470/505	[248]
Corn	GGCGCGAGGAAGGAGGUCUGAGGAGGUCACUGCGCC	DFHO (3,5-difluoro-4-hydroxybenzylidene imidazolinone-2-oxime)	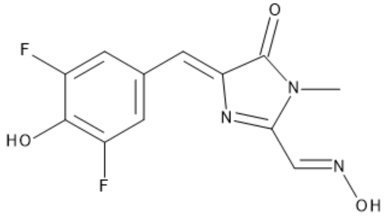	505/545	[208]
		DFAME (3,5-difluoro-4-hydroxybenzylidene imidazolinone-2-acrylate methyl)	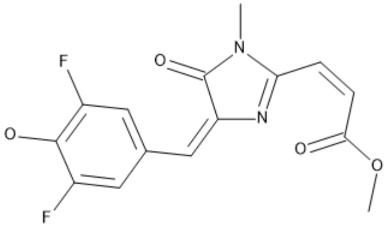	531/621	[249]
Mango I	GGCACGUACGAAGGGACGGUGCGGAGAGGAGAGUACGUGC	TO1-Biotin (tiazole orange 1-3PEG-biotin)	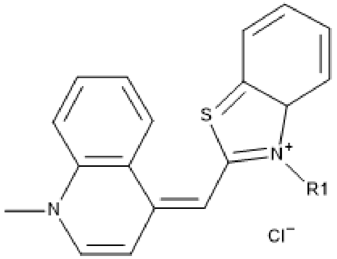	510/535	[209]

		TO3-Biotin (tiazole orange 3-3PEG-biotin)	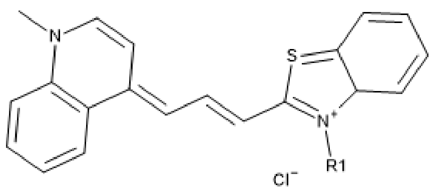	637/658	
Pepper	GGCGCACUGGCGCUGCGCCUUCGGGCGCCAAUCGUAGCGUGUCGGCGCC	HBC485 (4-[(Z)-1-cyano-2-[5-[2-(dimethylamino)ethyl-methyl-amino]pyrazin-2-yl]vinyl]benzonitrile)	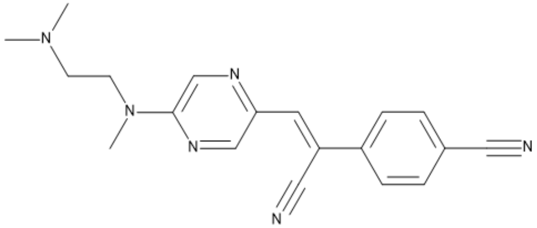	443/485	[210]
		HBC497 (4-[(Z)-1-cyano-2-[5-[2-hydroxyethyl(methyl)amino]pyrazin-2-yl]vinyl]benzonitrile)	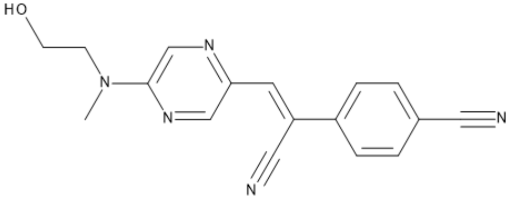	435/497	
		HBC508 (4-[(Z)-1-cyano-2-[6-[2-hydroxyethyl(methyl)amino]-3-pyridyl]vinyl]benzonitrile)	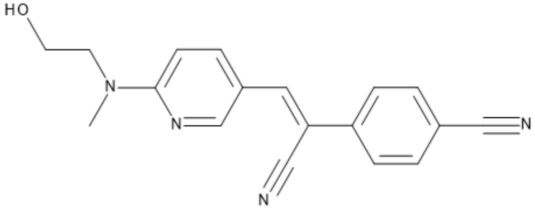	458/508	

		HBC514 (4-[(Z)-1-cyano-2-[4-[2-(dimethylamino)ethyl-methyl-amino]phenyl]vinyl]benzonitrile)	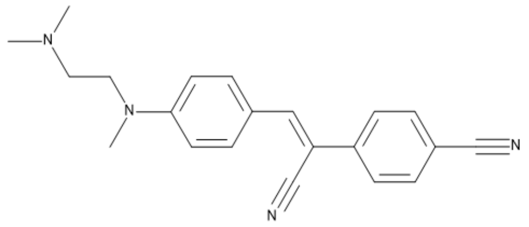	458/514	
		HBC525 ((E)-2-(1,3-benzoxazol-2-yl)-3-[4-[2-hydroxyethyl(methyl)amino]phenyl]prop-2-enenitrile	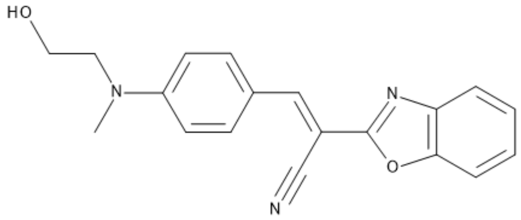	491/525	
		HBC530 (4-[(Z)-1-cyano-2-[4-[2-hydroxyethyl (methyl) amino]phenyl]vinyl] benzonitrile)	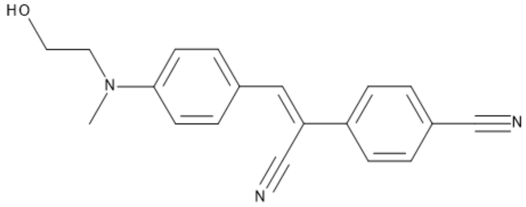	485/530	
		HBC599 (4-[(Z)-1-cyano-2-[6-[2-hydroxyethyl(methyl)amino]benzothiophen-2-yl]vinyl] benzonitrile)	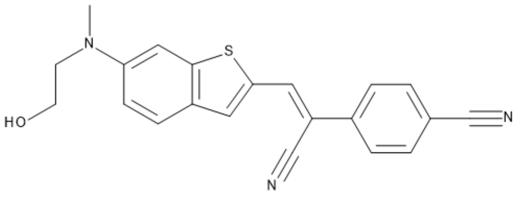	515/599	

		HBC620 (4-[(Z)-1-cyano-2-[5-[2-hydroxyethyl(methyl)amino]thieno[3,2-b]thiophen-2-yl]vinyl]benzonitrile	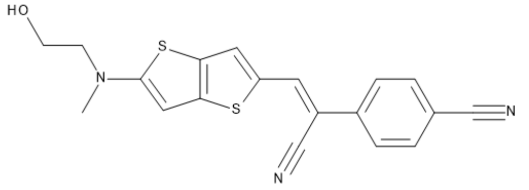	577/620	
Chili	GGCUAGCUGGAGGGGCGCCAGUUCGCUGGUGGUUGGGUGCGGUCGGCUAGCC	DMHBI ((Z)-5-(4-Hydroxy-3,5-dimethoxybenzylidene)-2,3-dimethyl-3,5-dihydro-4H-imidazol-4-one)	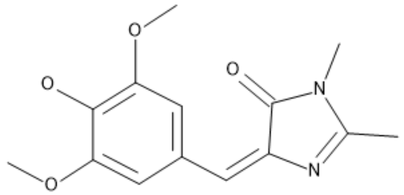	400/537	[250]
		DMHBI+ ([4-[(4E)-4-[(4-hydroxy-3,5-dimethoxy-phenyl)methylene]-1-methyl-5-oxo-imidazol-2-yl]phenyl]-trimethyl-ammonium iodide)	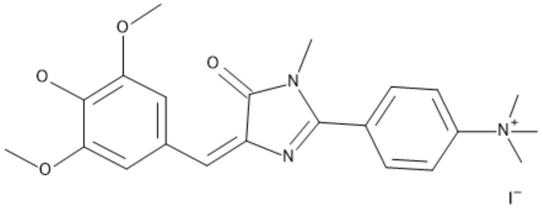	413/542	
		DMHBO+ ([(Z)-4-(3,5-Difluoro-4-hydroxy-benzylidene)-5-oxo-1-(4-(trimethylammonium)phenyl)-4,5-dihydro-1H-imidazole-2- carbaldehyde oxime iodide)	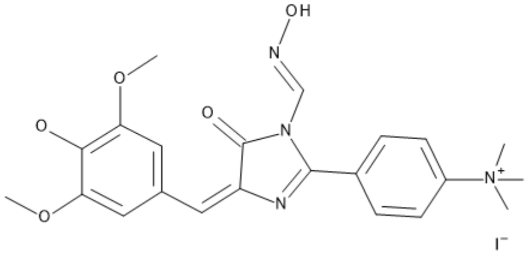	456/592	

Okra	CGGGGACCAUCCAGCGAU GGAUACUGCUGCUUCGGC AGAGCAAGGAUAAUAUC UAGAACGACCAUCCG	ACE (3-(2,3,6,7-tetrahydro-1H,5H-pyrido(3,2,1-ij)quinolin-9-yl)acrylamide-2-cyano-N-(2-(2-aminoethoxy) ethyl))	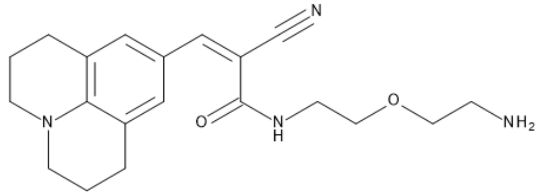	468/505	[251]
Squash	GGGAAGAUACAAGGUGA GCCCAAUAAUAUGGUUUG GGUUAGGAUAGGAAGUA GAGCCUUAAACUCUCUAA GCGGUAUCUUCCC	DFHBI-1T ((Z)-4-(3,5-difluoro-4-hydroxybenzylidene)-2-methyl-1-(2,2,2-trifluoroethyl)-1H-imidazol-5(4H)-one)	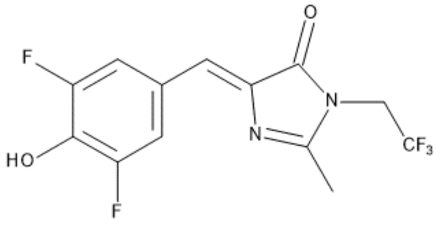	452/503	[252]
		DFHO (3,5-difluoro-4-hydroxybenzylidene imidazolinone-2-oxime)	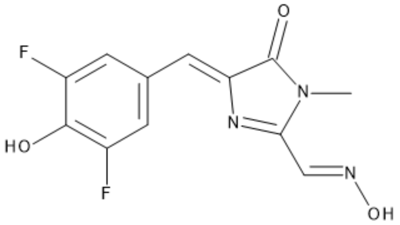	495/562	
		DFQL-1T ((5Z)-5-[(3,5-difluoro-4-hydroxyphenyl)methylene]-3,5-dihydro-2-methyl-3-(2,2,2-trifluoroethyl)-4H-imidazol-4-one)	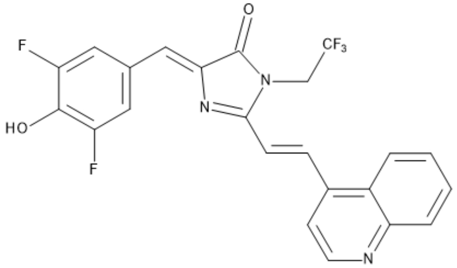	516/654	[226]

Beetroot	GCGCCGGUUAGGCAGAGGUGGGUGGUGUGGAGGAGUAUCUGUCCGGCGC	DFAME (3,5-difluoro-4-hydroxybenzylidene imidazolinone-2-acrylate methyl)	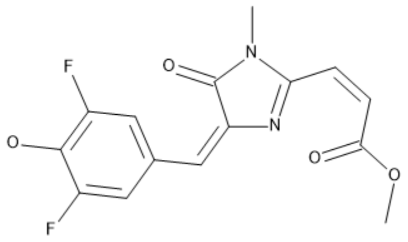	514/619	[249]
		DFHO (3,5-difluoro-4-hydroxybenzylidene imidazolinone-2-oxime)	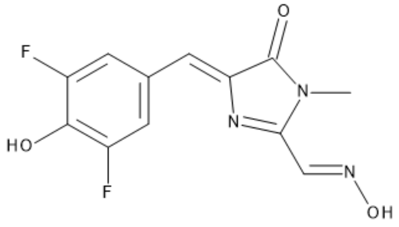	473/551	
Clivias	GAAGAUUGUAAACAUGCCGAAAGGCAGACACUUCC	NBSI (4-(N-(2-hydroxyethyl)(methyl))benzylidene-3-methyl-2-styryl-3,5-dihydro-4H-imidazol-4-one)	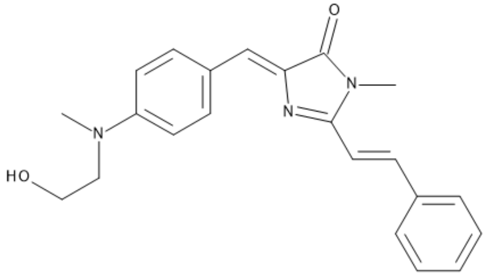	524/580	[253]
Peach I	GGAAUCCCUGAAGGAUGUGGGGGAGGGAGAAGGGAUUCC	TO3-Biotin (tiazole orange 3-3PEG-biotin)	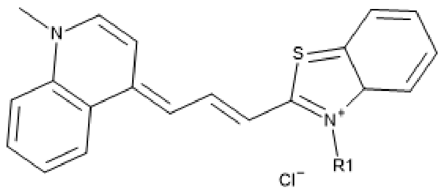	637/658	[254]

DIR2s-Apt	GGAUGCGCCUUGAAAAGCCUGCGAAACACGCAGCUGGUGAAUGACAGCUAUGGCGCAUCC	DIR (dimethylindole red)	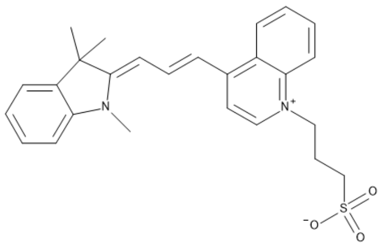	600/646	[255]
SRB-2	GGAACCUCGCUUCGGCGAUGAUGGAGAGGCGCAAGGUUAACCGCCUCAGGUUCC	SR-DN (sulforhodamine B dinitroaniline)	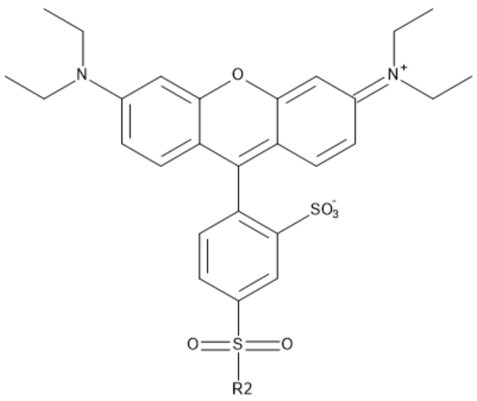	579/596	[256]
		TMR-DN (tetramethylrhodamine-dinitroaniline)	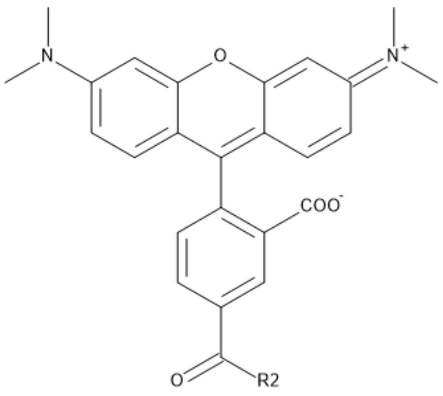	579/596	

DNB	GGUGCCUUAUUCCGGACGCCGGGCCCGAAUGCUGCUACGGCAGUCGAAGACAACAUCGCGCCCUUCGGAGGCACC	SR-DN (sulforhodamine B dinitroaniline)	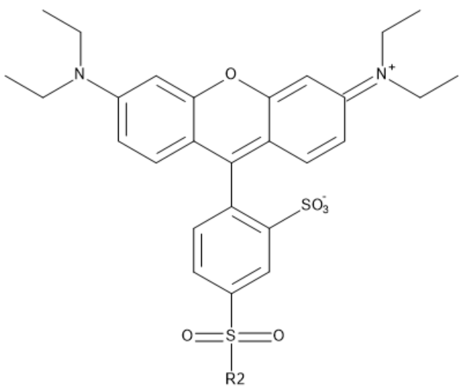	572/591	[257]
		FL-DN (fluorescein-dinitroaniline)	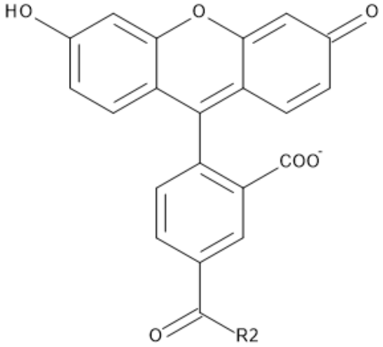	498/528	
		RG-DN (rhodamine green-dinitroaniline)	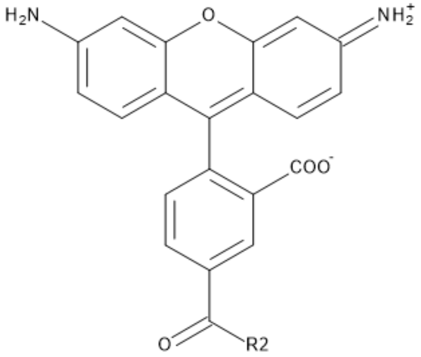	507/534	

		TMR-DN (tetramethylrhodamine-dinitroaniline)	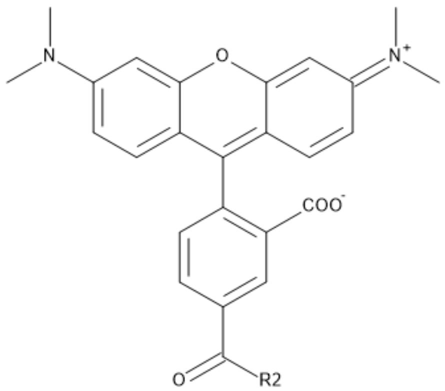	555/582	
		TR-DN (Texas Red-dinitroaniline)	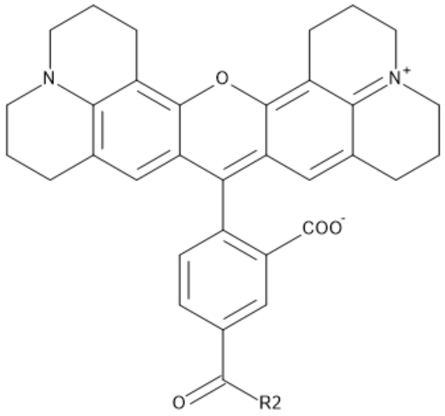	598/619	

RhoBAST	GGAACCUCCGCGAAAGCGGUGAAGGAGAGGCGCAAGGUUAACCGCCUCAGGUUCC	TMR-DN (tetramethylrhodamine-dinitroaniline)	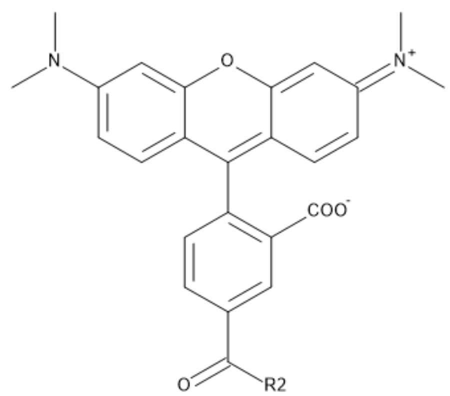	564/590	[215]
o-Coral	GGGAGACAGCUAGAGUACAGAACCCCGCUUCGGCGGUGAUGGAGAGGCGCAAGGUUAACCGCCUCAGGUUCCGGUGACGGGGCCUCGCUUCGGCGAUGAUGGAGAGGCGCAAGGUUAACCGCCUCAGGUUCUGACACGAGCACAGUGUAC	Gemini-561 (Sulforhodamine B dimer-PEG3-biotin)	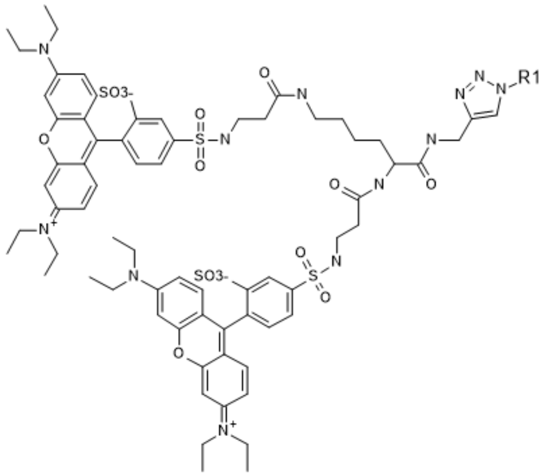	580/596	[258]

BeCA	GGAGGUACGUGGACUGCUUAUGCAGUAGGAAGGCCUCC	BC6 (benzopyrylium and coumarin derivate)	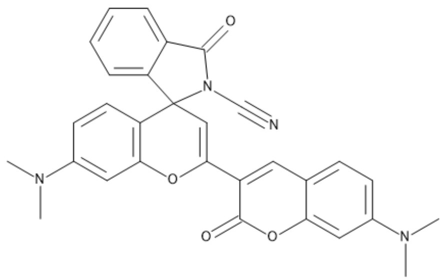	665/684	[216]
SiRA	GGCCACCGGGUUUGAAAACCUGGCUGCUUCGGCAGUUGUAUCCUUUGGCC	SiR (silicon rhodamine)	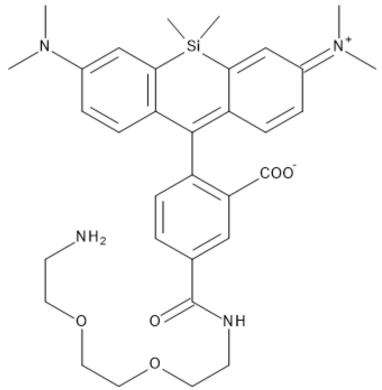	649/662	[259]
DNA aptamers					
DAP1042	CAATTACGGGGGAGGGTGTGTGGTCTTGCTTGGTTCGTATTG	DSEDA (dapoxyl sulfonyl ethylenediamine)	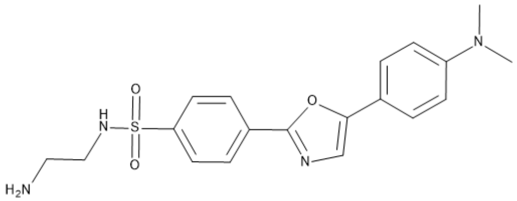	402/540	[260]
		DSA (dapoxyl sulfonic acid)	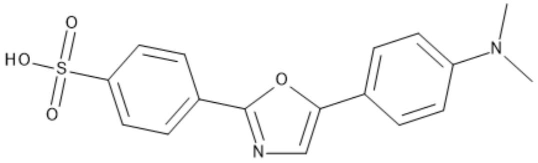	391/497	

02a	TATGTGGAGGGCAAGAGG GTGTTGATGGGGTCGGTGG GTCCTGCCCTCTCATCG	TO1-Biotin (tiazole orange 1-3PEG-biotin)	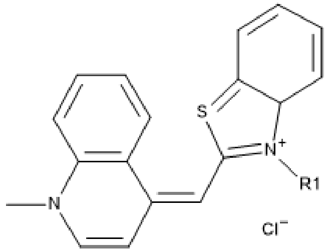	488/535	[213]
Lettuce	AACGTGCTCAAGGTGTCTT AGTAGGGATGATGCGGCA GTGGGCTTCGCAGAACAG TGTTTATTCTGCGAGGGGA CTAAGACTCCTTCAGCAAG TTAAGC	DFHBI-1T ((Z)-4-(3,5-difluoro-4-hydroxybenzylidene)-2-methyl-1-(2,2,2-trifluoroethyl)-1H-imidazol-5(4H)-one)	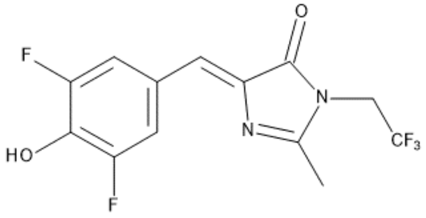	455/510	[212]
DIR2-1	GACGACGACGCTAGGAAG GCGTTGGTGGGCACGCCG GTCGTC	DIR (dimethylindole red)	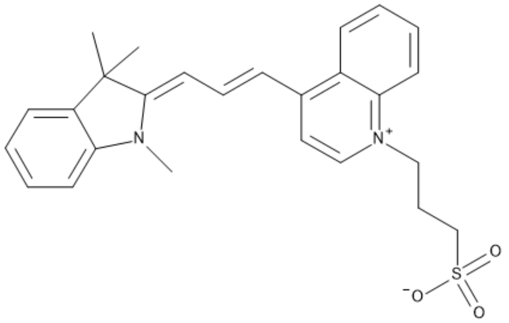	602/650	[261]

Hoechst_class_I-1-mini	CAATTCGGGATAAAGGGGGCCATTG	Hoechst derivate 6 (2,6-Diisopropyl-4-(5-(4-methylpiperazin-1-yl)-1H,1′H-2,5′-bibenzo[d]imidazol-2′-yl)phenol)	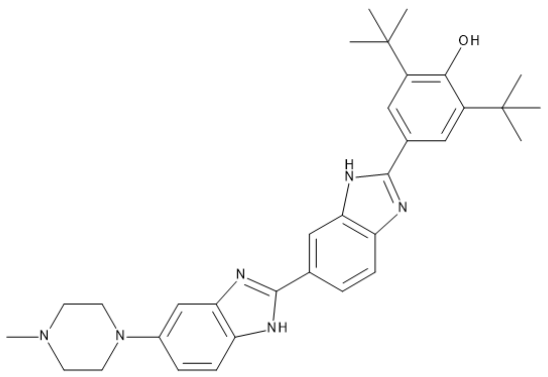	345/460	[262]
CV30S	AACGACCACCGGTGCGCCGTACAGGTAACTAGCGTCGTCGTT	CV (Crystal Violet)	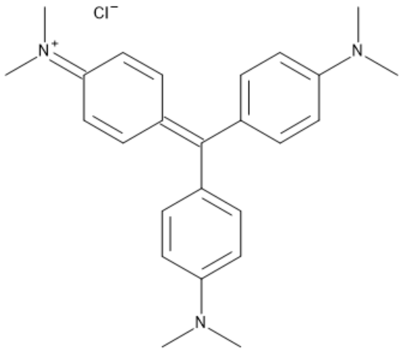	580/640	[214]
R1 = 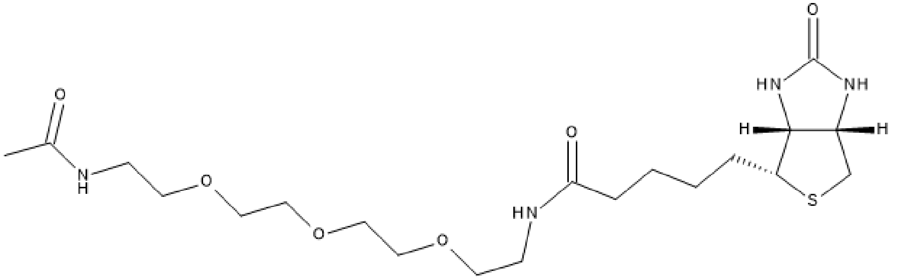			R2 = 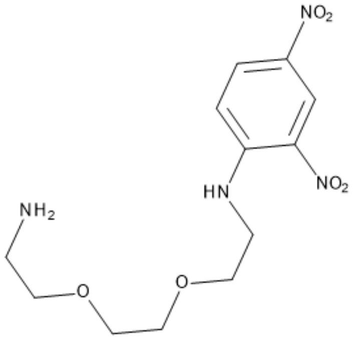		
